# Supplementary material for: Peptidoglycan-Chi3l1 interaction shapes gut microbiota in intestinal mucus layer
Source: eLife. 2024 Oct 7;13:RP92994. doi: 10.7554/eLife.92994 (PMC11458176; doi:10.7554/eLife.92994)

Raw unedited membranes

Figure 1—Source Data 1

Figure 1C

Cropped and labelled membranes

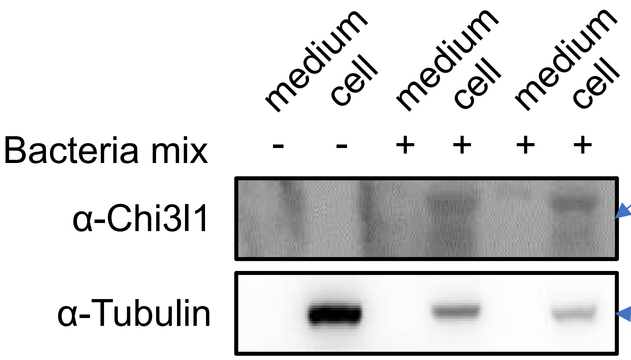

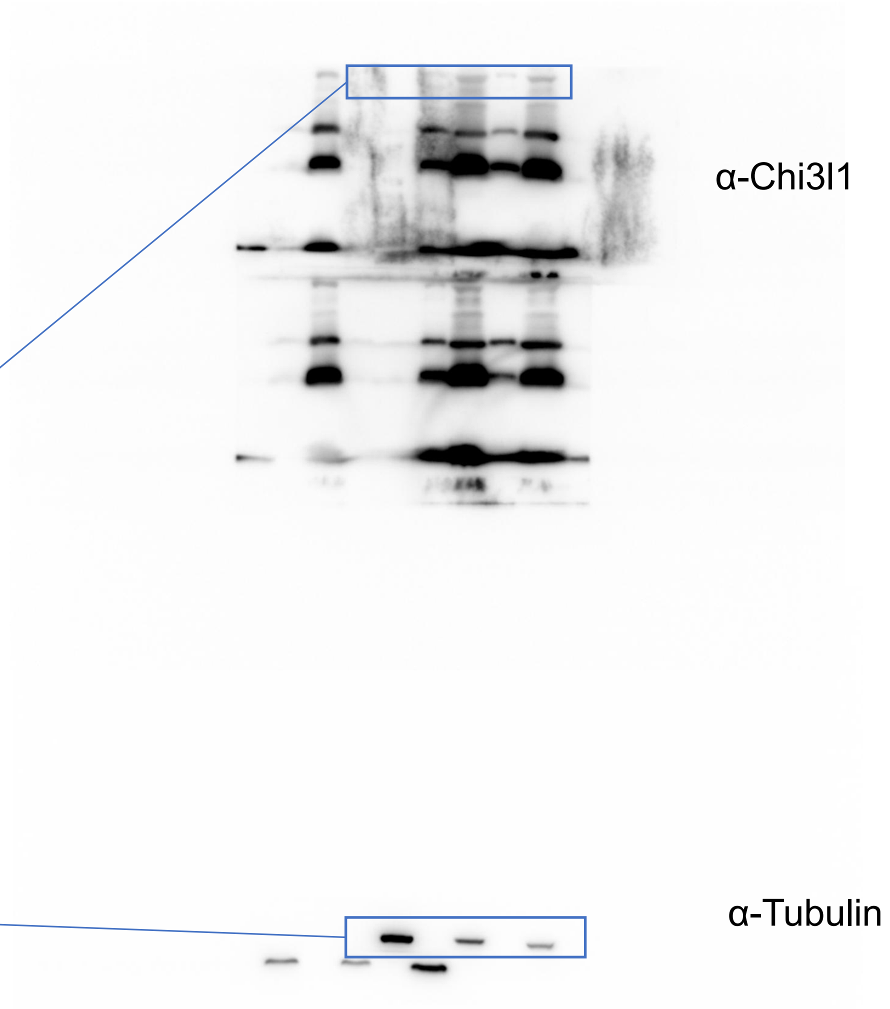

Figure 1—Source Data 1

Figure 1D

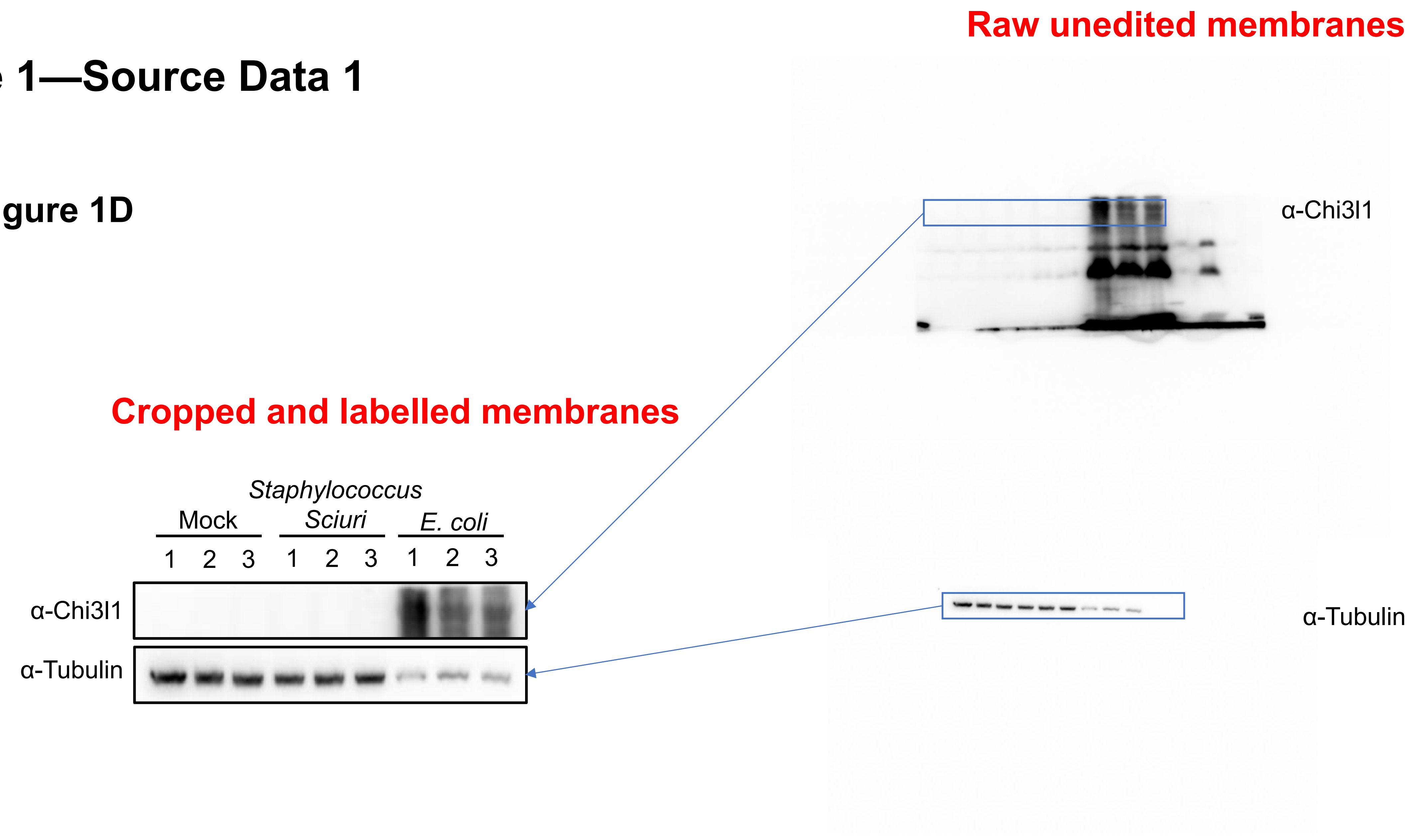

Figure 1—Source Data 1

Figure 1E

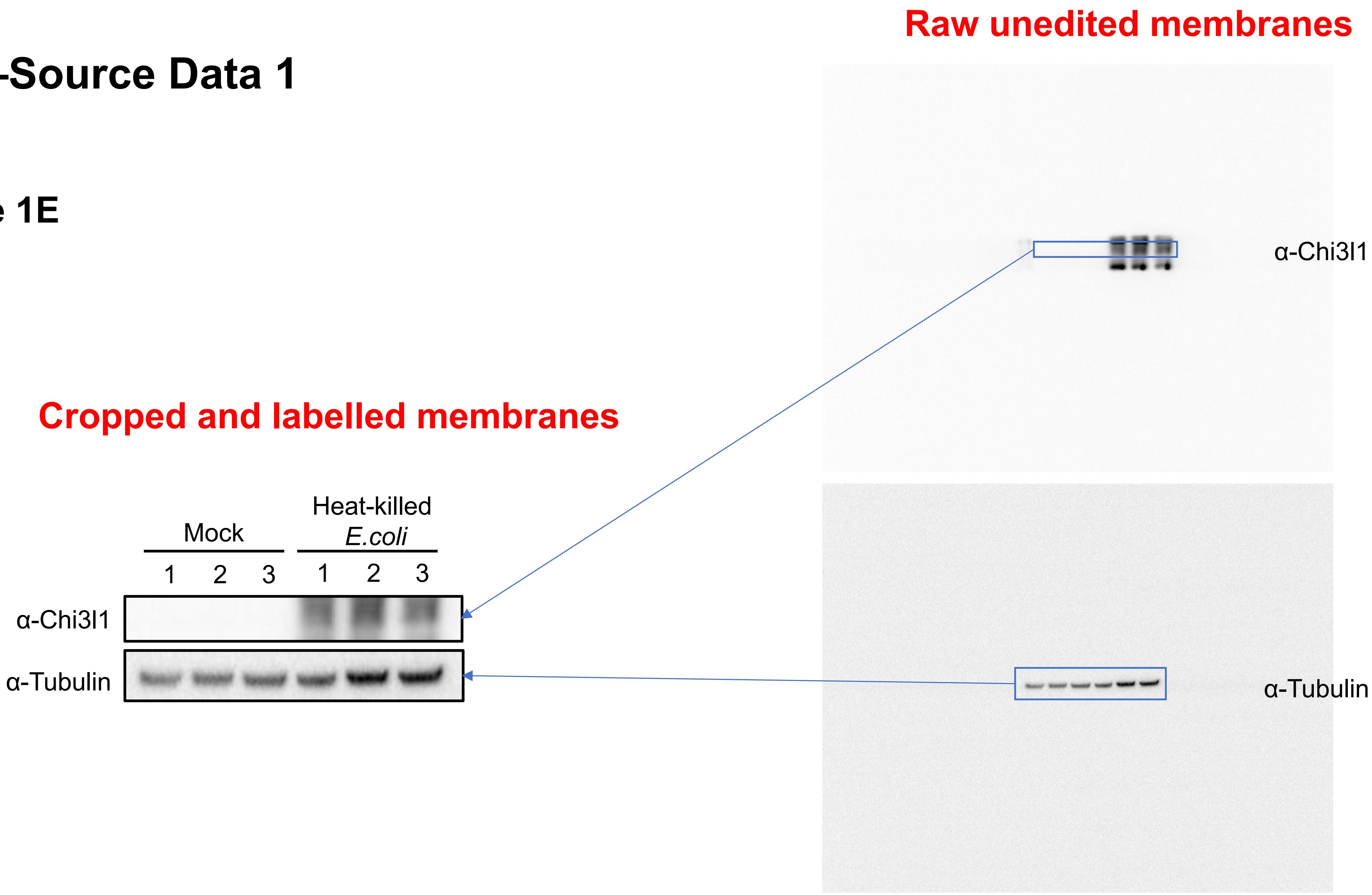

Figure 1—Source Data 1

Figure 1F

Cropped and labelled membranes

Raw unedited membranes

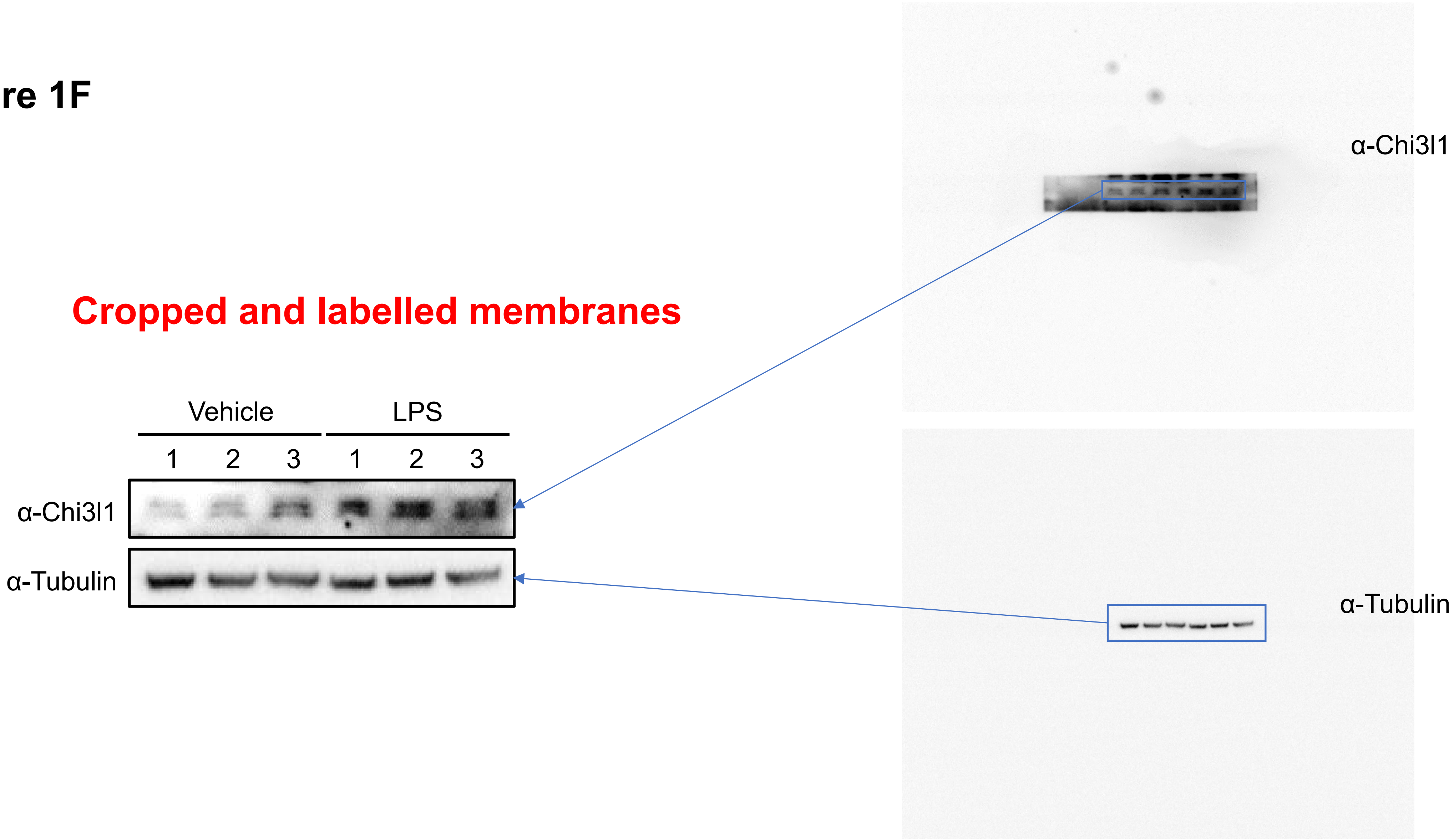

Supplement: Figure 1—source data 1. [file elife-92994-fig1-data1.zip › Figure 1-source data 1.pdf]
