# Supplementary figures and images for: Peptidoglycan-Chi3l1 interaction shapes gut microbiota in intestinal mucus layer
Source: eLife. 2024 Oct 7;13:RP92994. doi: 10.7554/eLife.92994 (PMC11458176; doi:10.7554/eLife.92994)

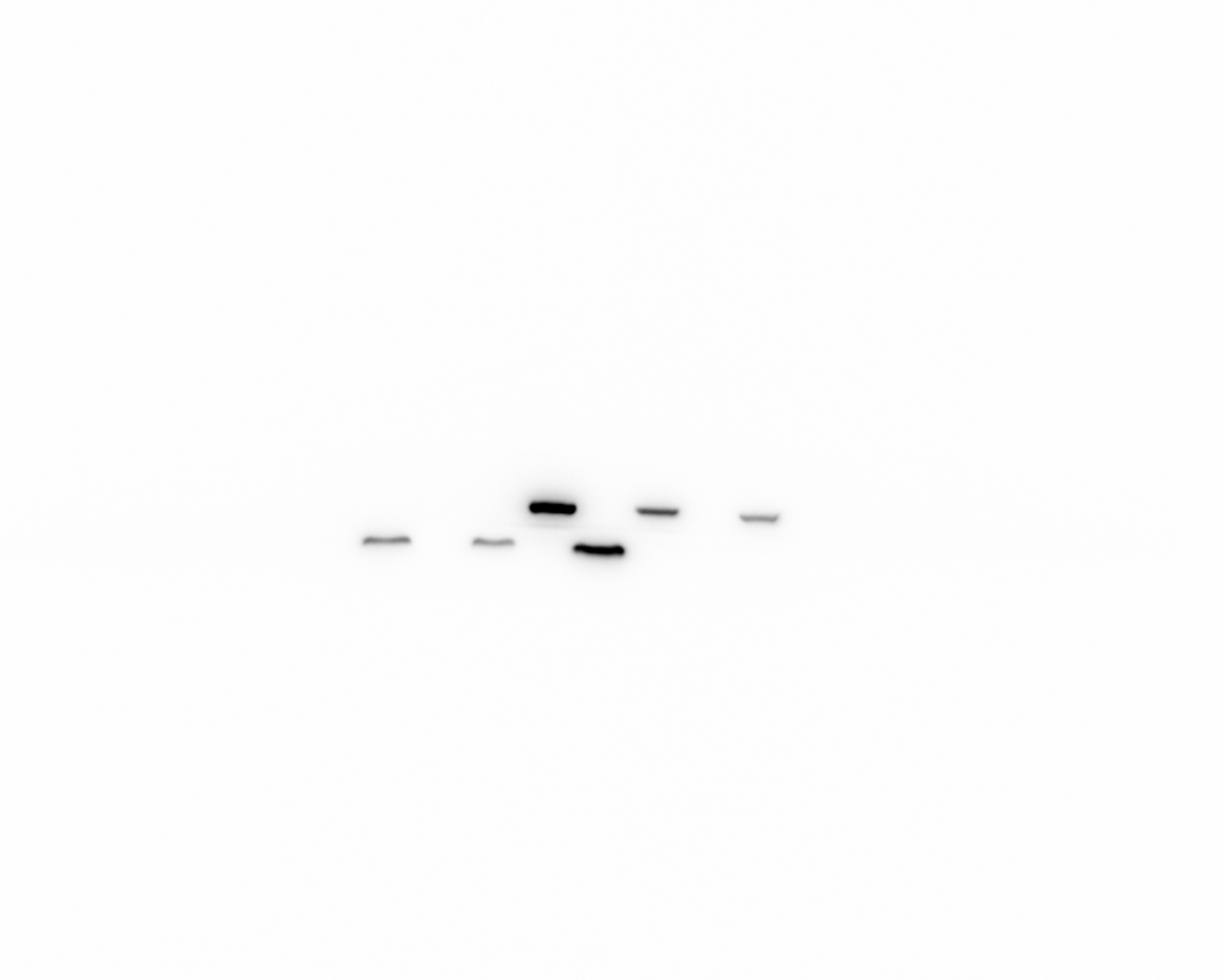

Supplement: Figure 1—source data 2. [file elife-92994-fig1-data2.zip › Figure 1C-1.tif]

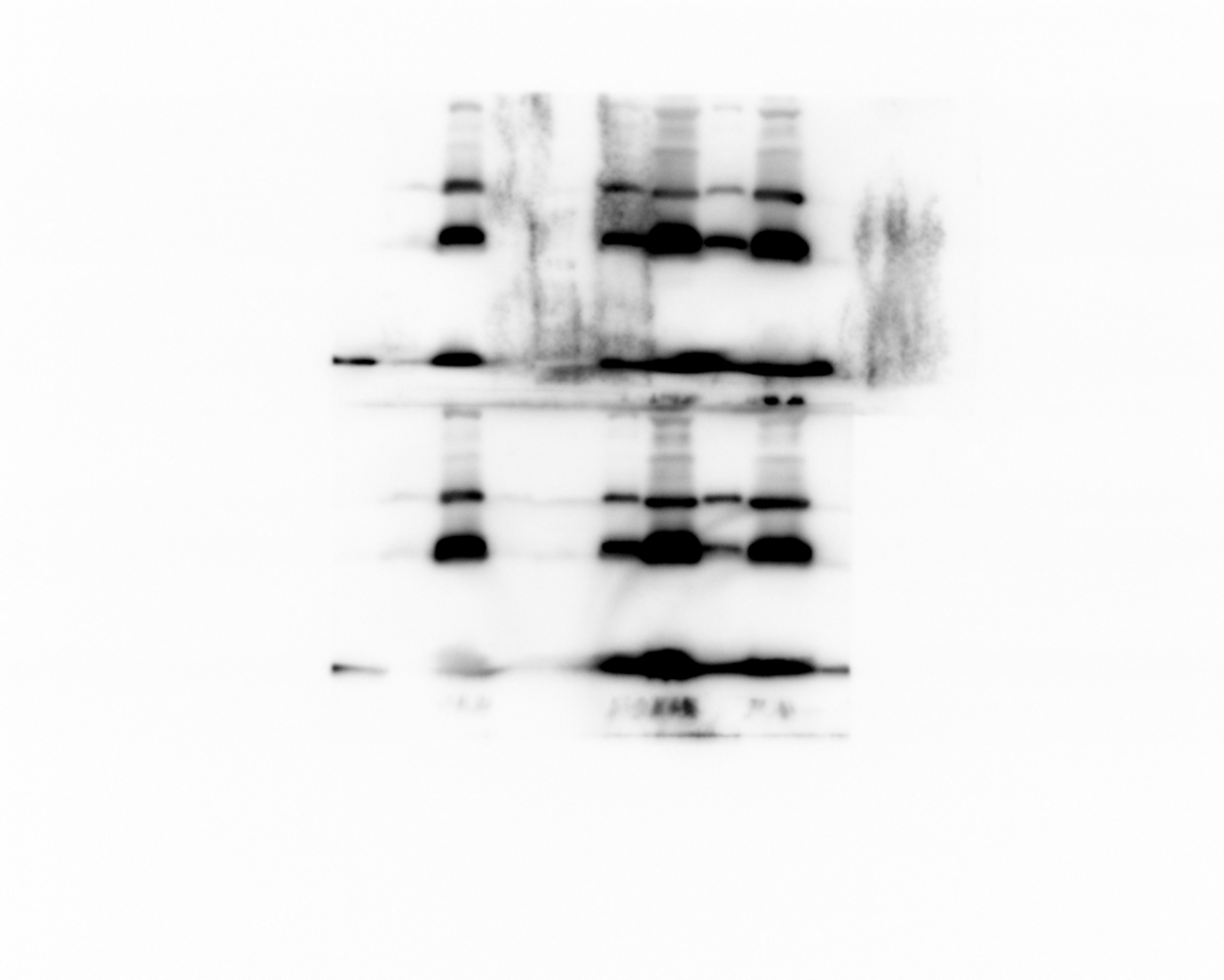

Supplement: Figure 1—source data 2. [file elife-92994-fig1-data2.zip › Figure 1C-2.tif]

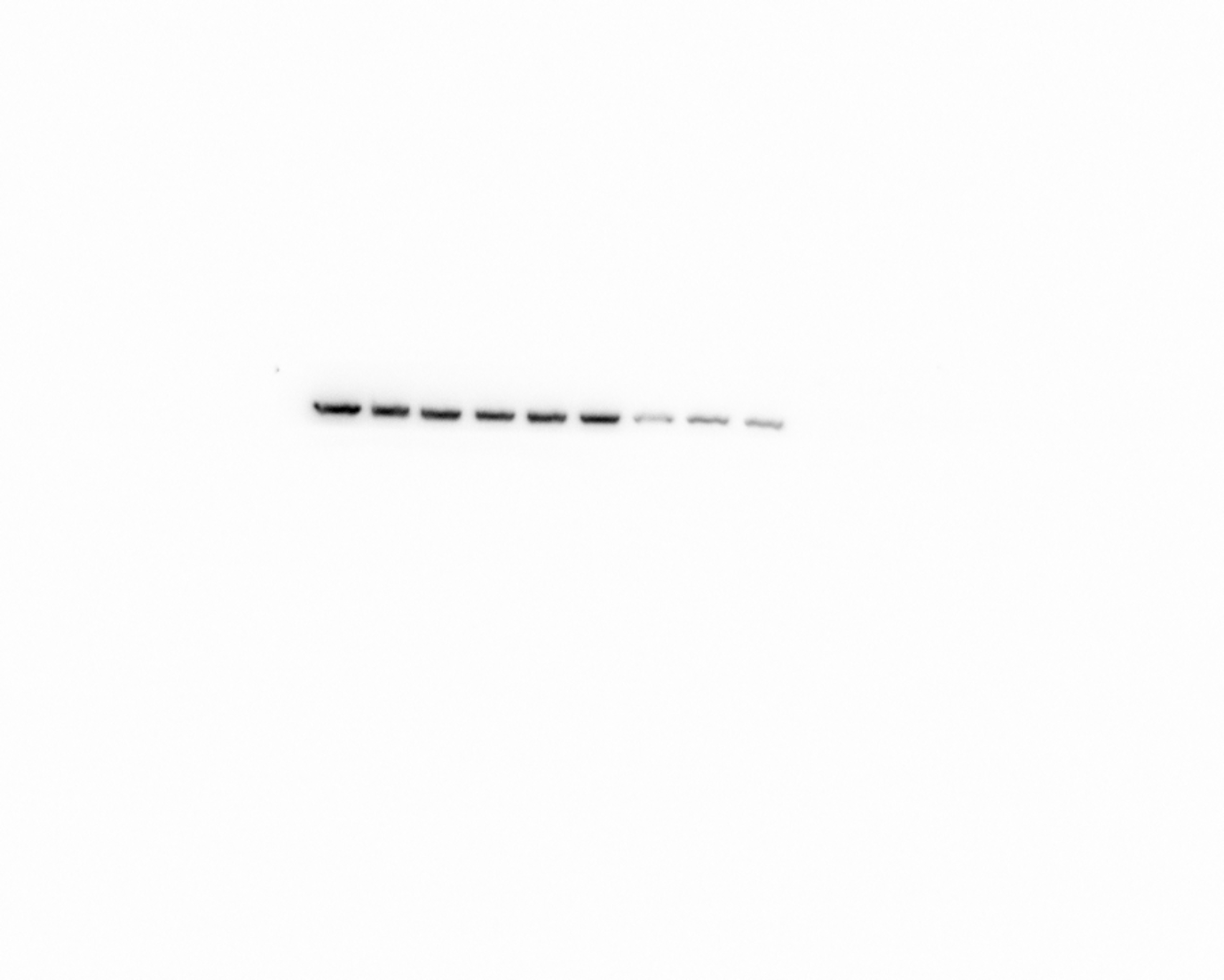

Supplement: Figure 1—source data 2. [file elife-92994-fig1-data2.zip › Figure 1D-1.tif]

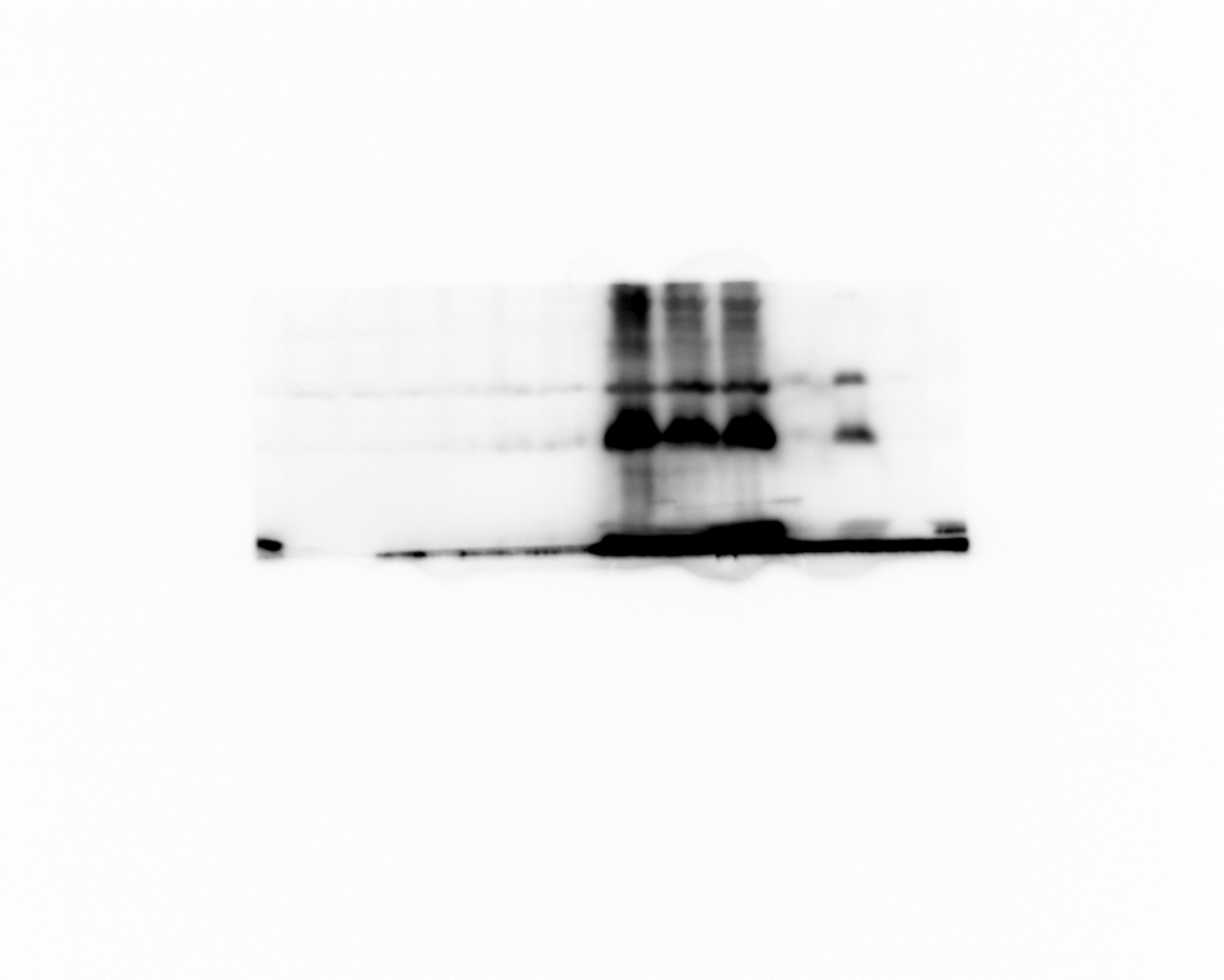

Supplement: Figure 1—source data 2. [file elife-92994-fig1-data2.zip › Figure 1D-2.tif]

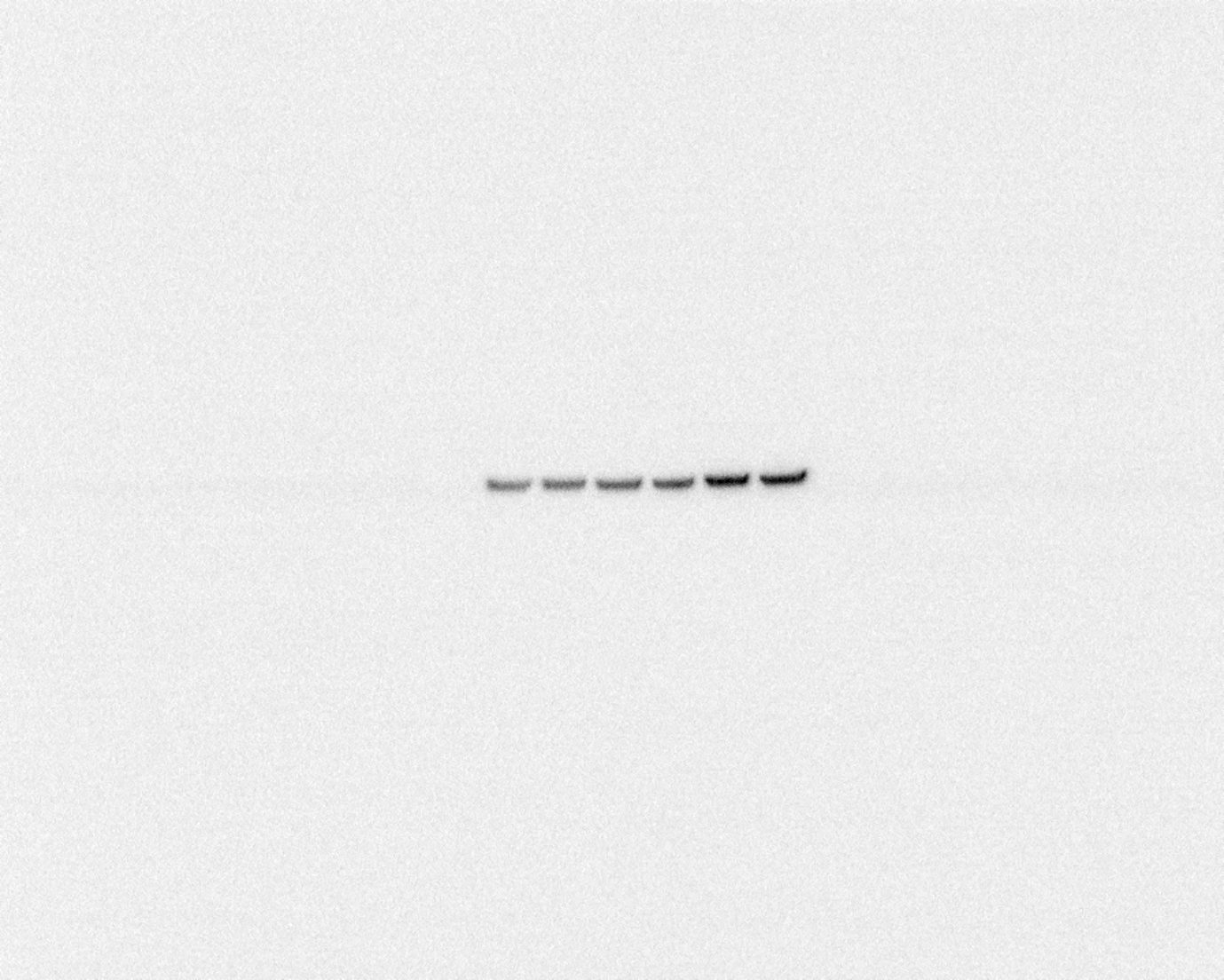

Supplement: Figure 1—source data 2. [file elife-92994-fig1-data2.zip › Figure 1E-1.tif]

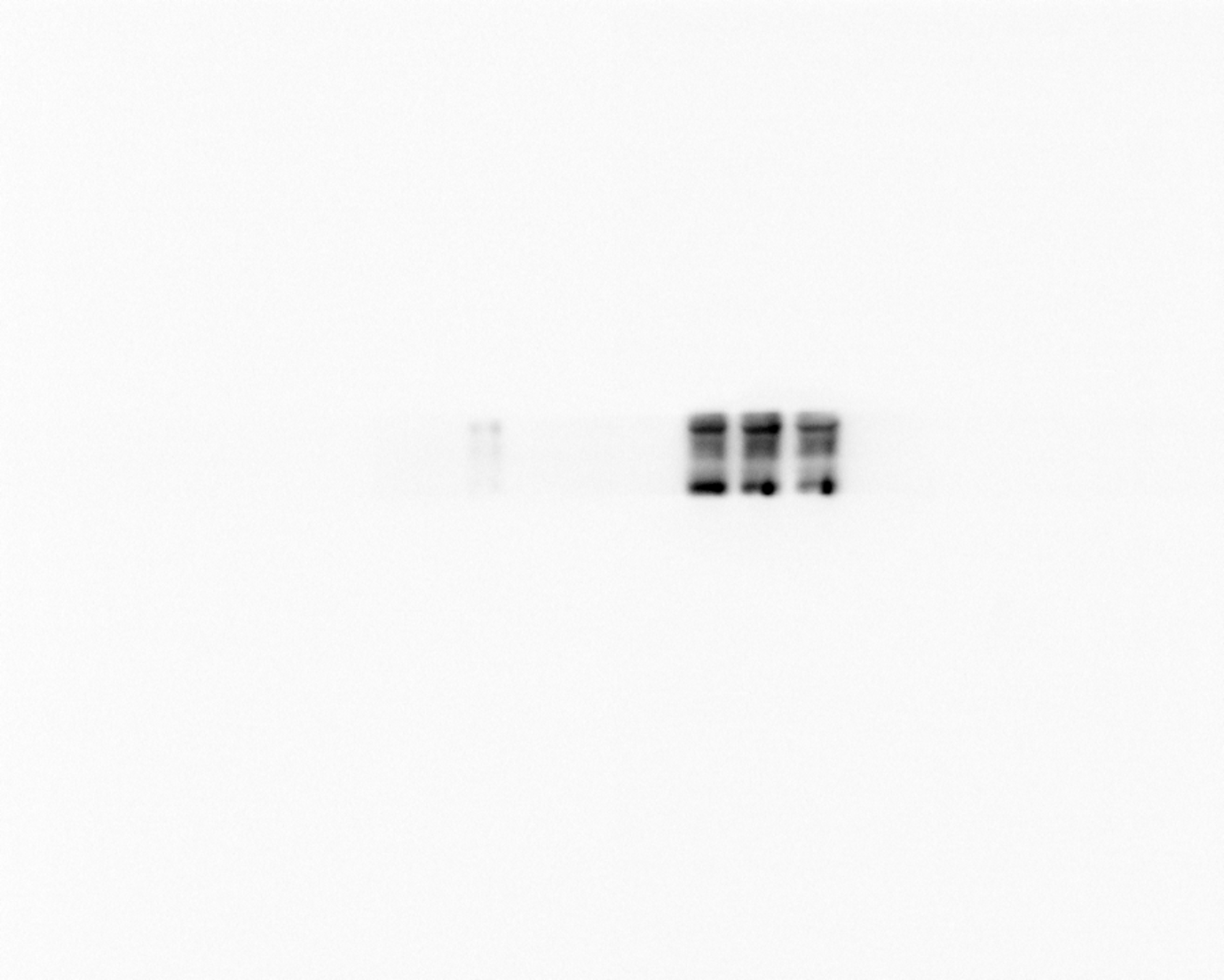

Supplement: Figure 1—source data 2. [file elife-92994-fig1-data2.zip › Figure 1E-2.tif]

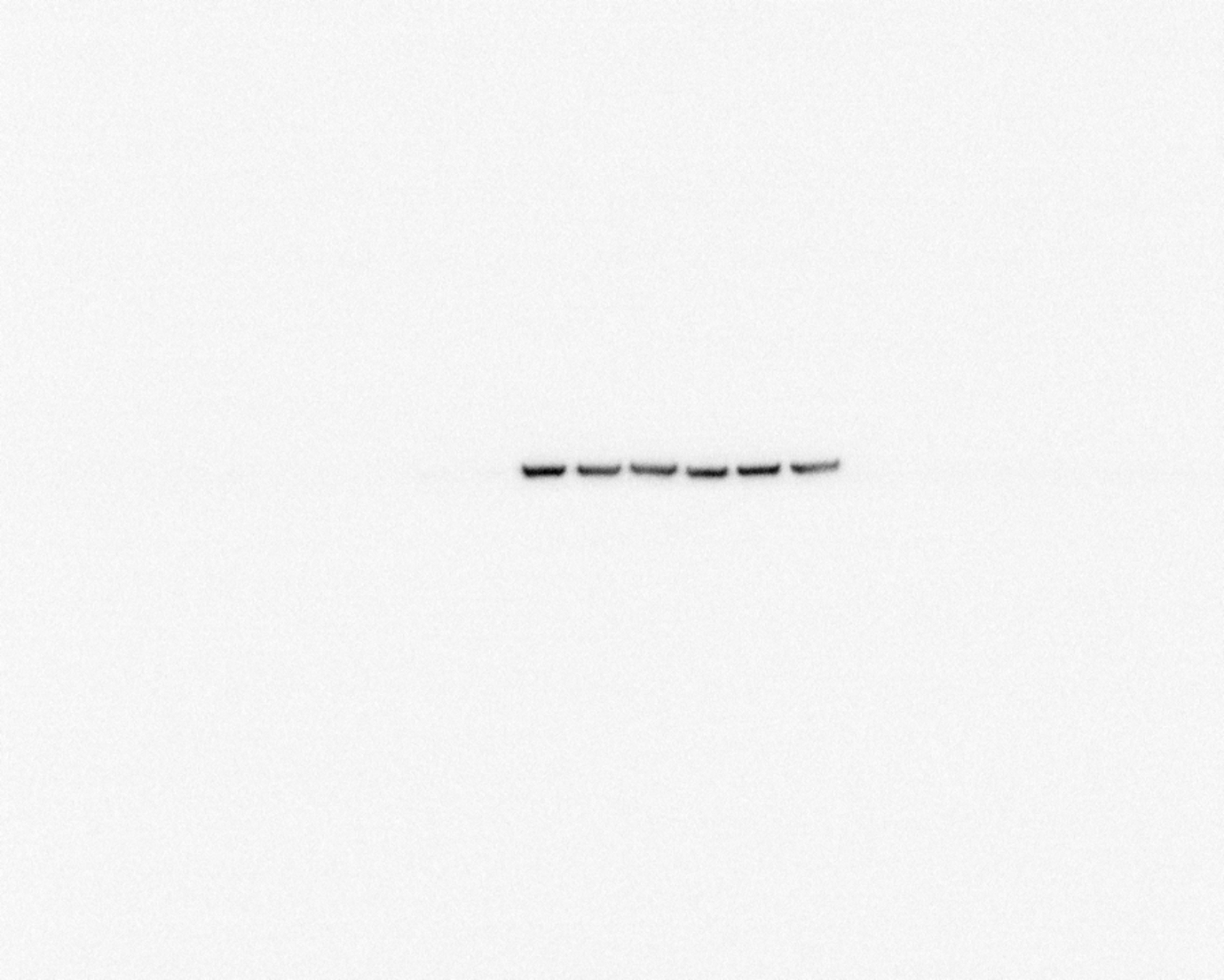

Supplement: Figure 1—source data 2. [file elife-92994-fig1-data2.zip › Figure 1F-1.tif]

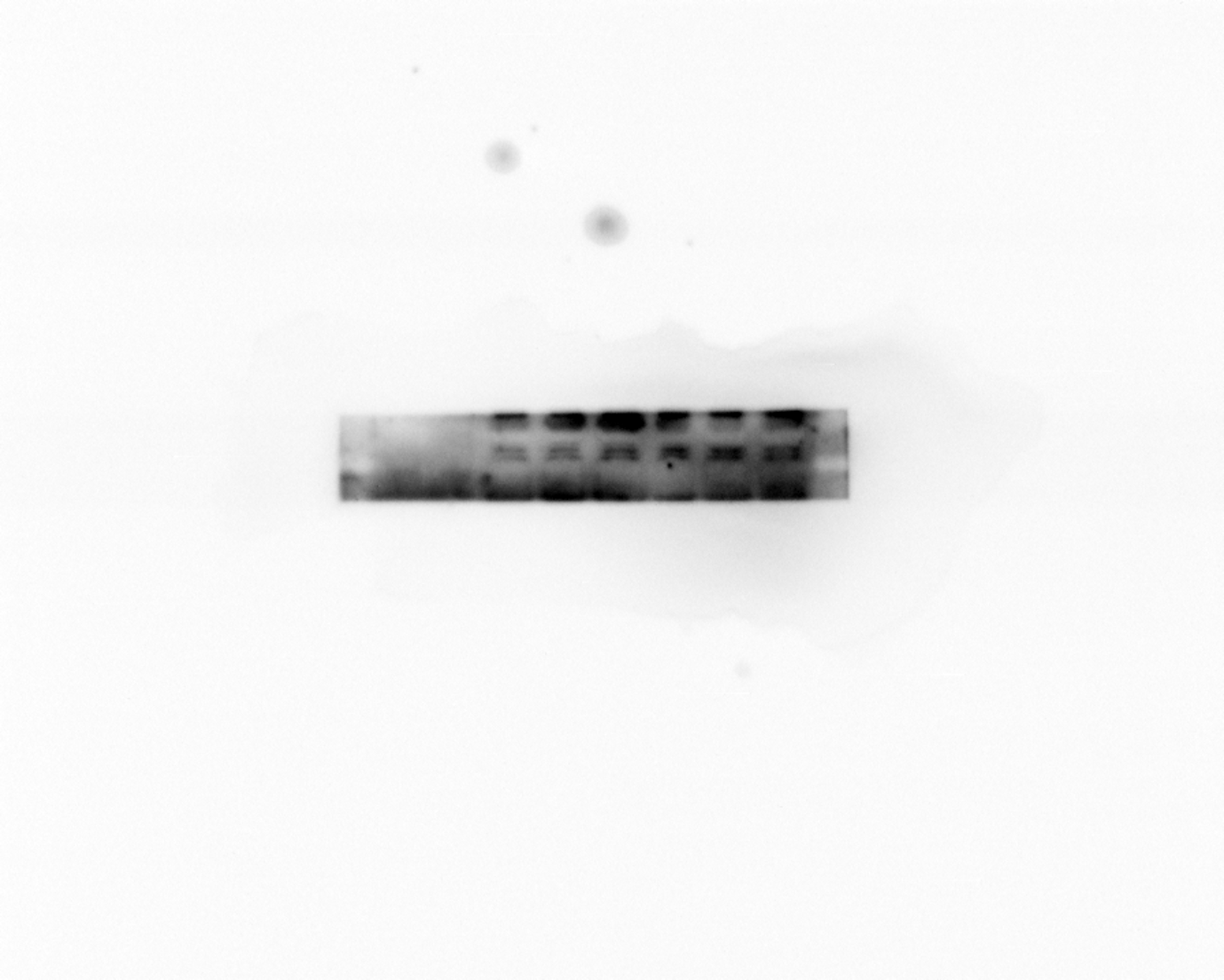

Supplement: Figure 1—source data 2. [file elife-92994-fig1-data2.zip › Figure 1F-2.tif]

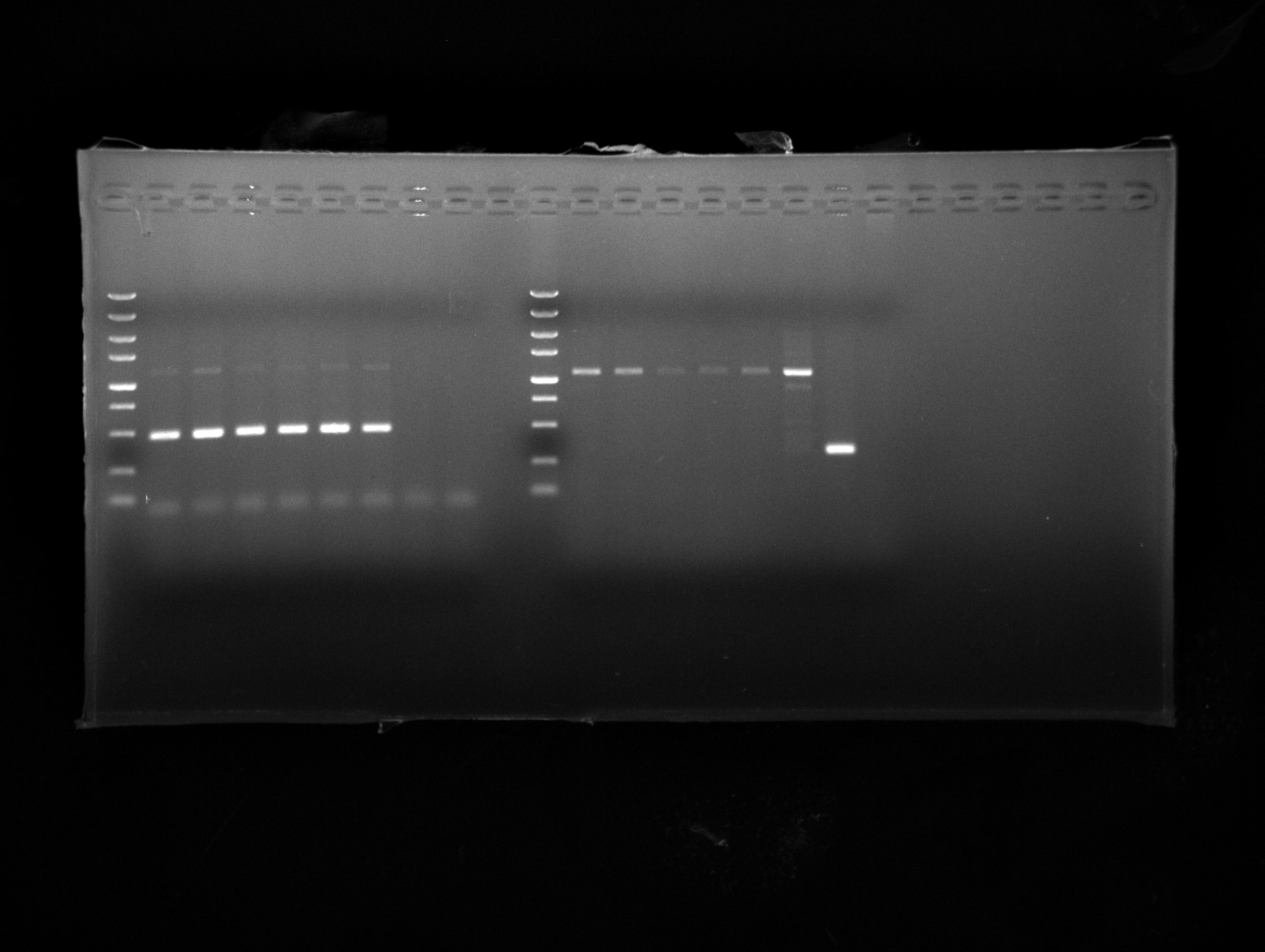

Supplement: Figure 1—figure supplement 1—source data 2. [file elife-92994-fig1-figsupp1-data2.zip › Figure 1ΓÇöfigure supplement 1-source data 2.tif]

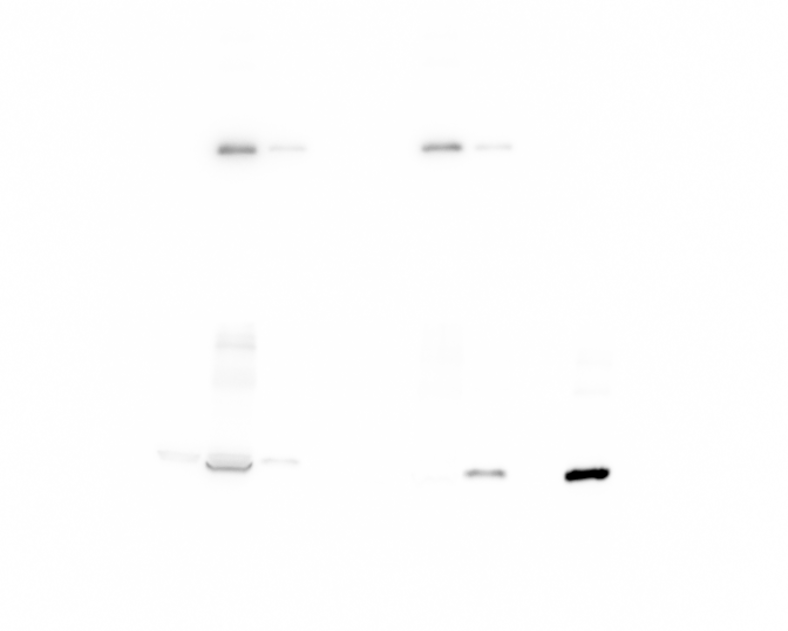

Supplement: Figure 2—source data 2. [file elife-92994-fig2-data2.zip › Figure 2-source data 2/Figure 2B.tif]

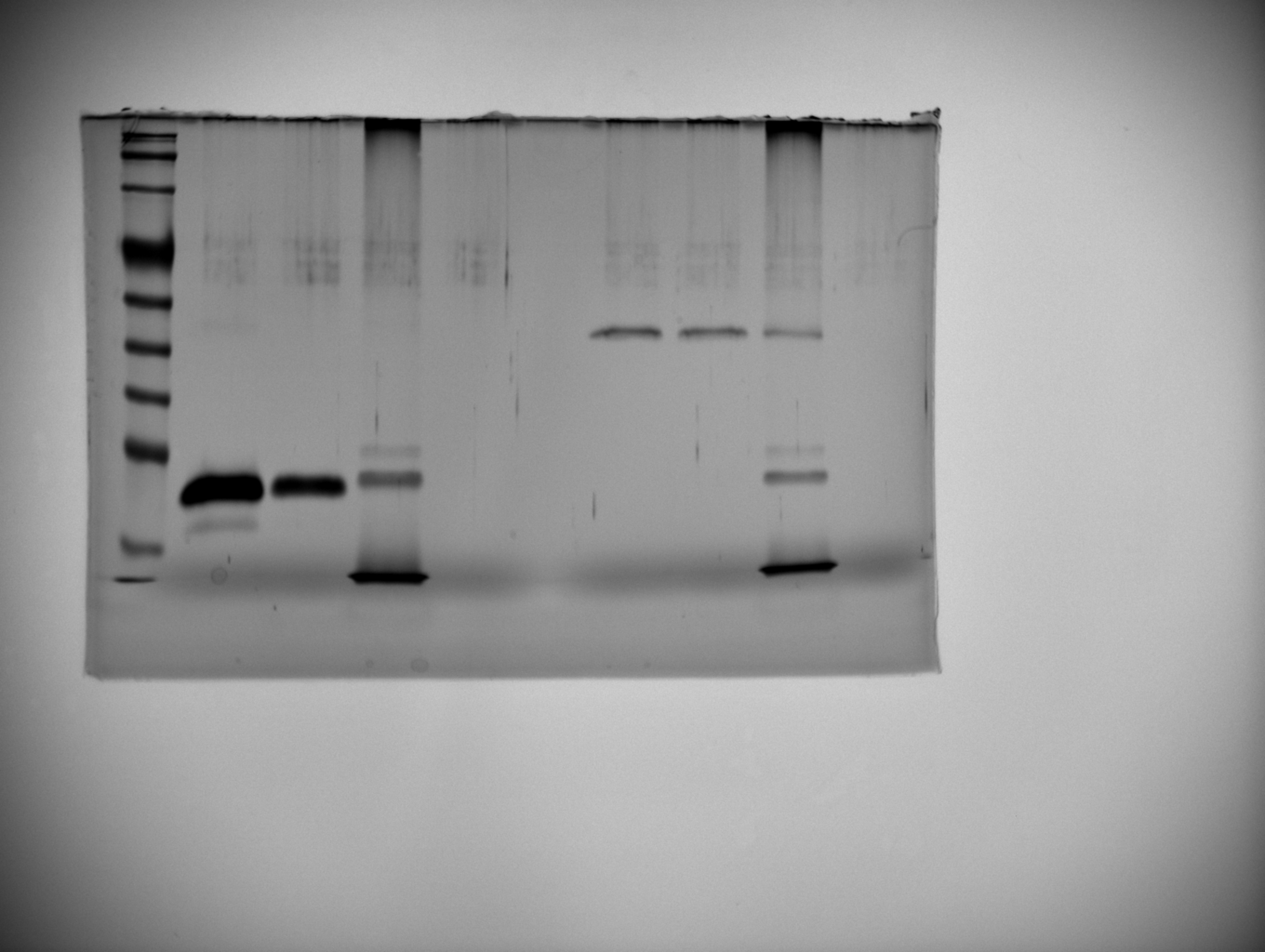

Supplement: Figure 2—source data 2. [file elife-92994-fig2-data2.zip › Figure 2-source data 2/Figure 2C-1.tif]

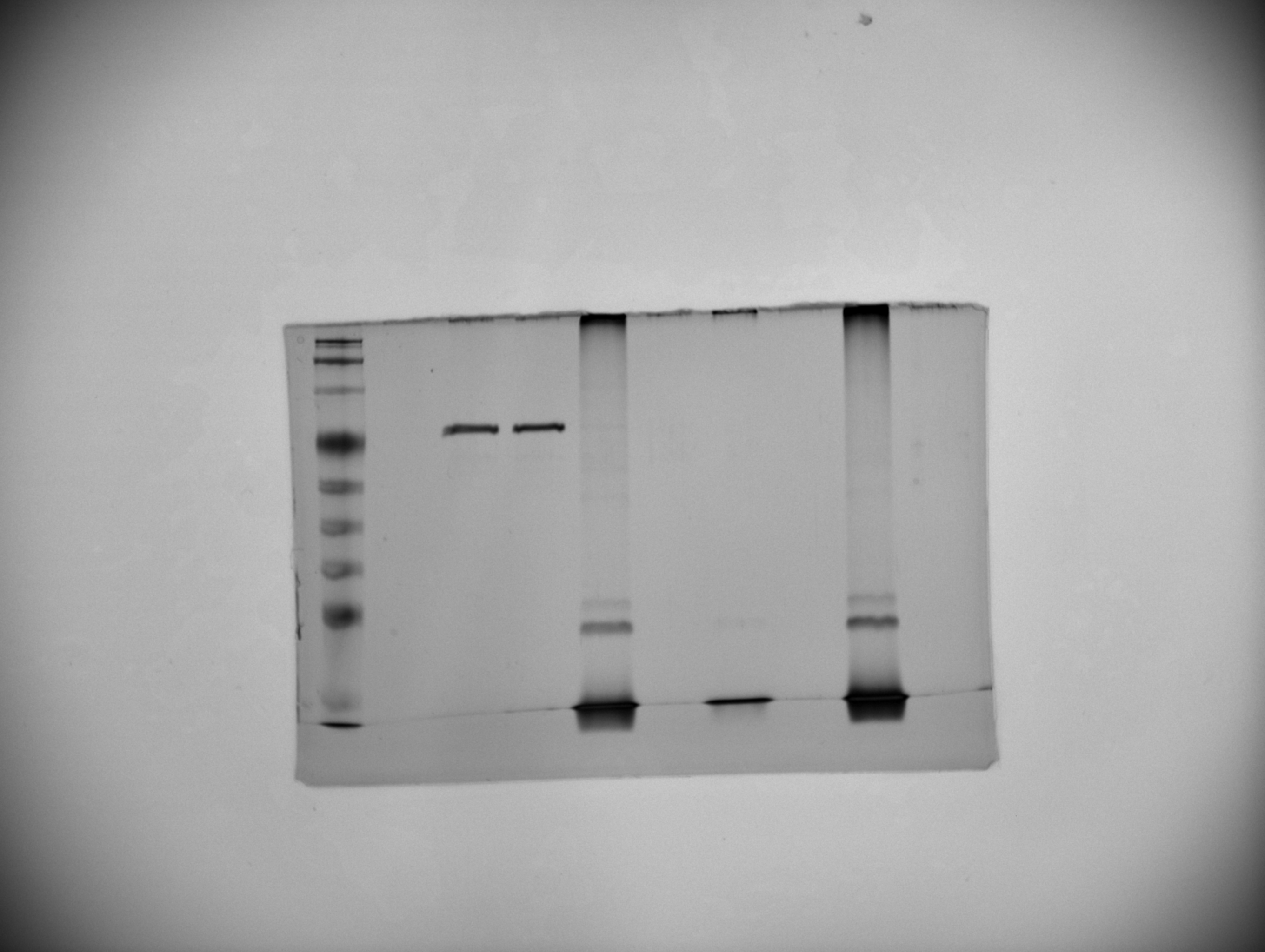

Supplement: Figure 2—source data 2. [file elife-92994-fig2-data2.zip › Figure 2-source data 2/Figure 2C-2.tif]

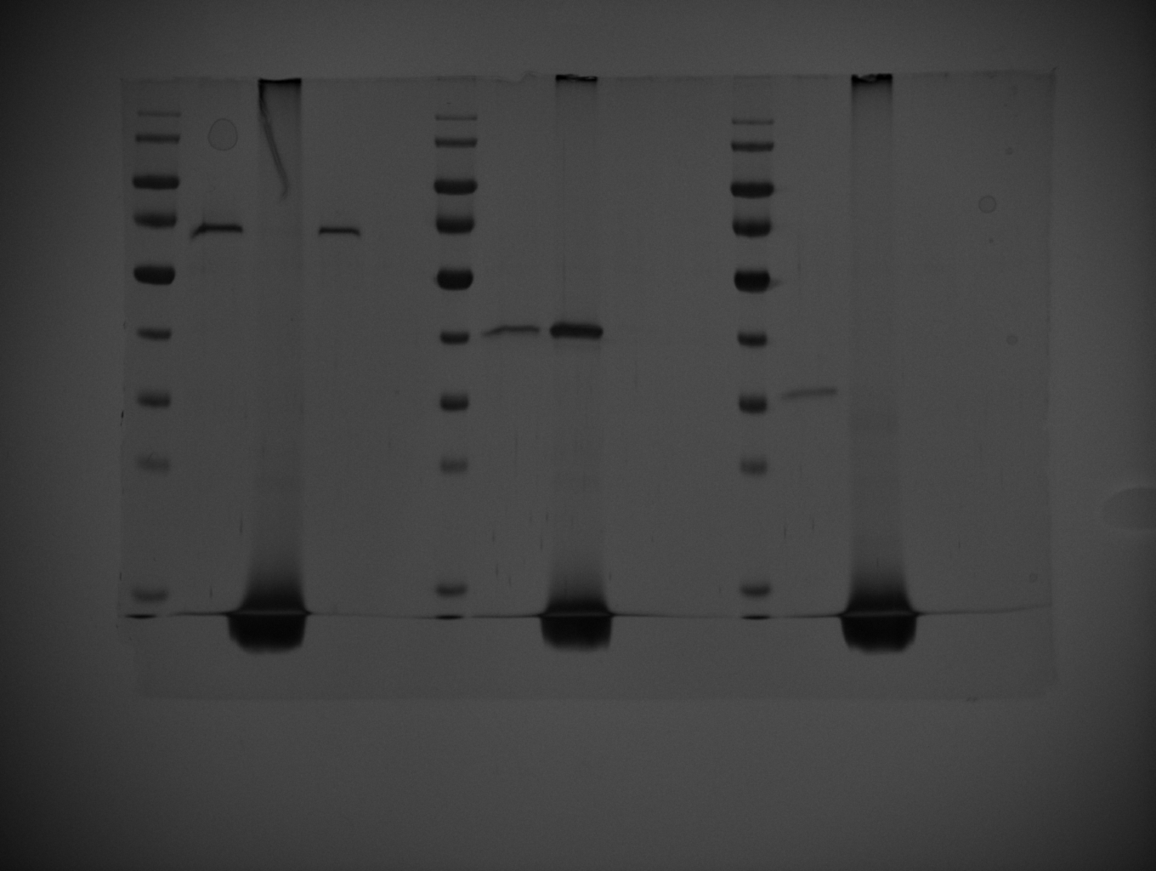

Supplement: Figure 2—source data 2. [file elife-92994-fig2-data2.zip › Figure 2-source data 2/Figure 2D.tif]

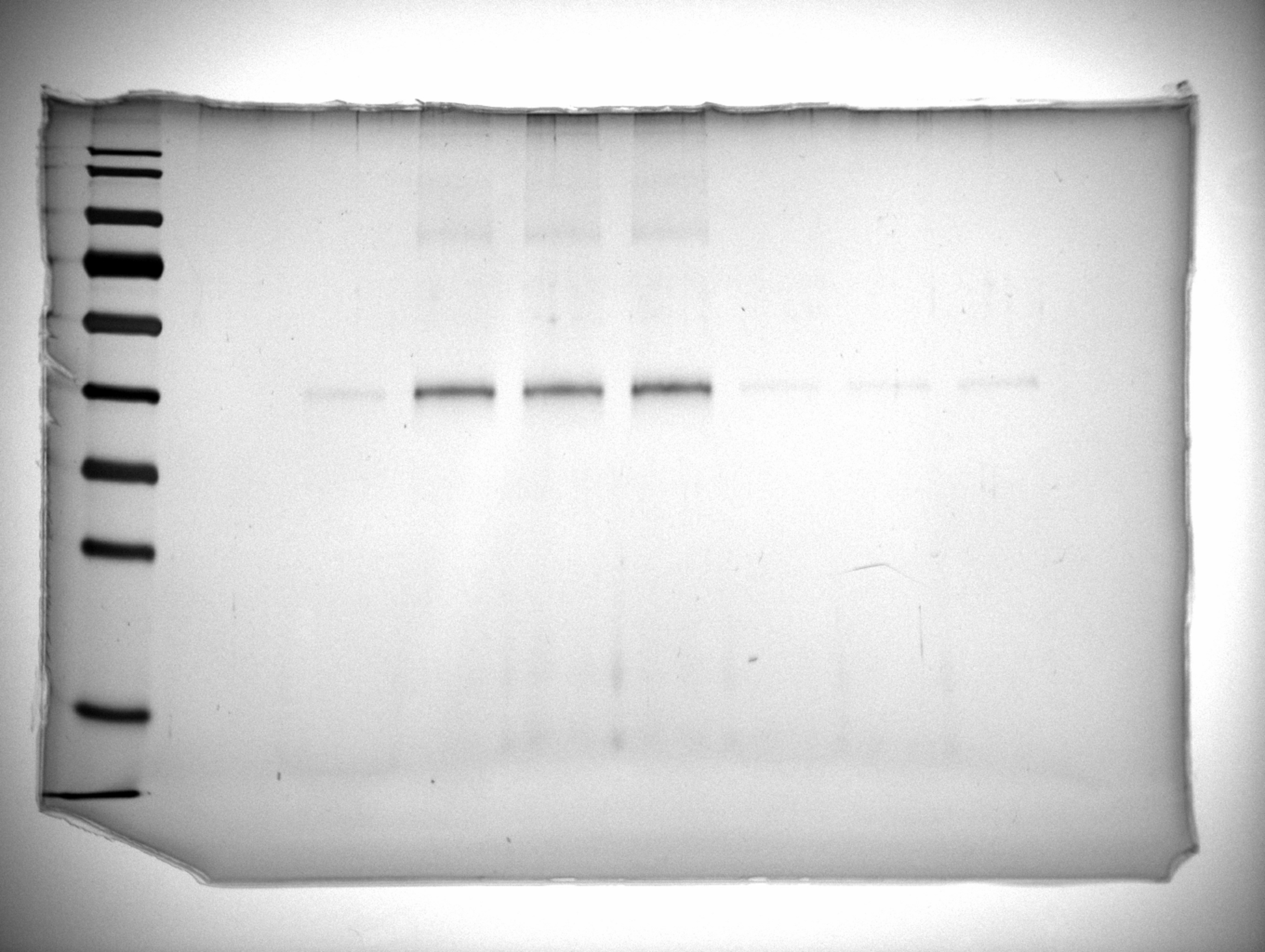

Supplement: Figure 2—source data 2. [file elife-92994-fig2-data2.zip › Figure 2-source data 2/Figure 2E-1.tif]

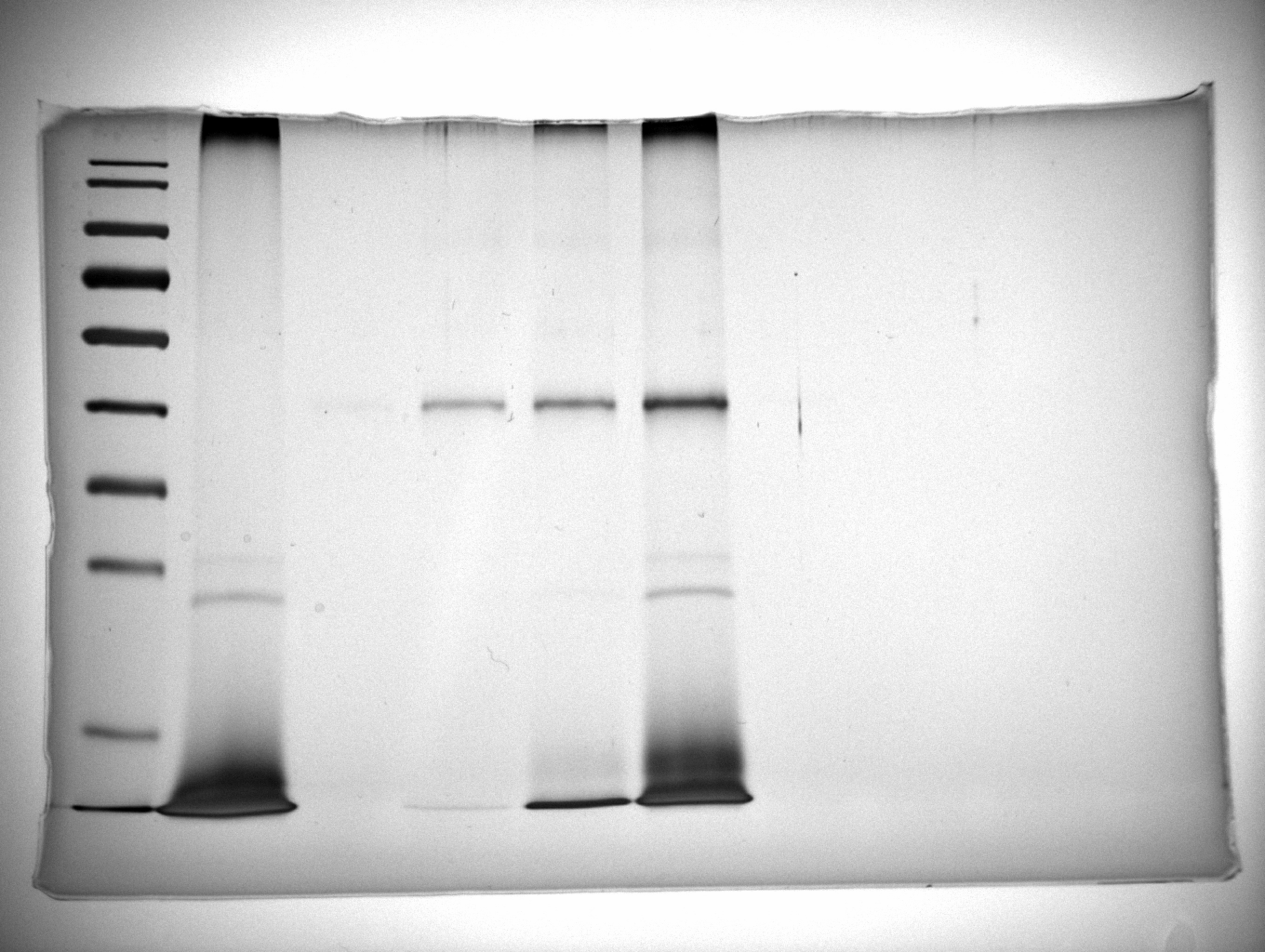

Supplement: Figure 2—source data 2. [file elife-92994-fig2-data2.zip › Figure 2-source data 2/Figure 2E-2.tif]

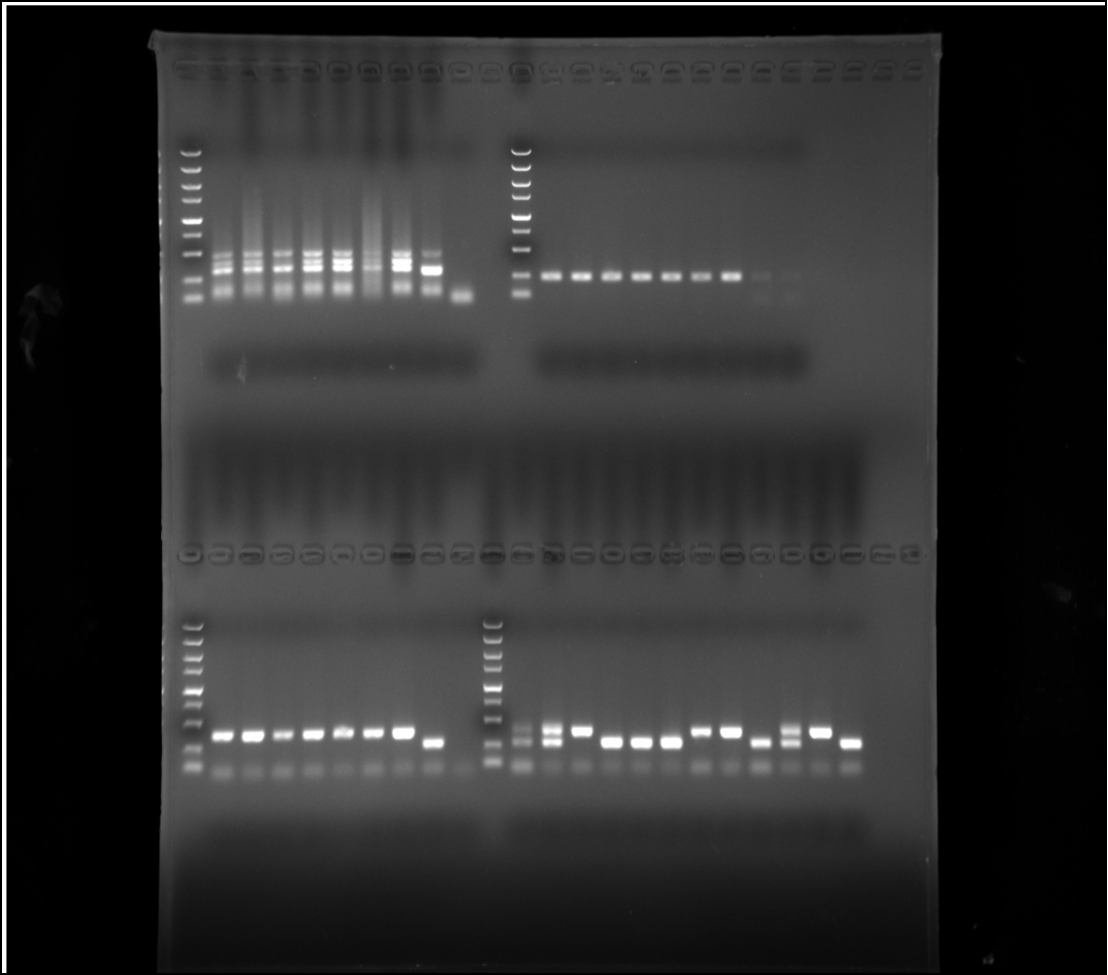

Supplement: Figure 3—figure supplement 1—source data 2. [file elife-92994-fig3-figsupp1-data2.zip › Figure 3ΓÇöfigure supplement 1B.tif]

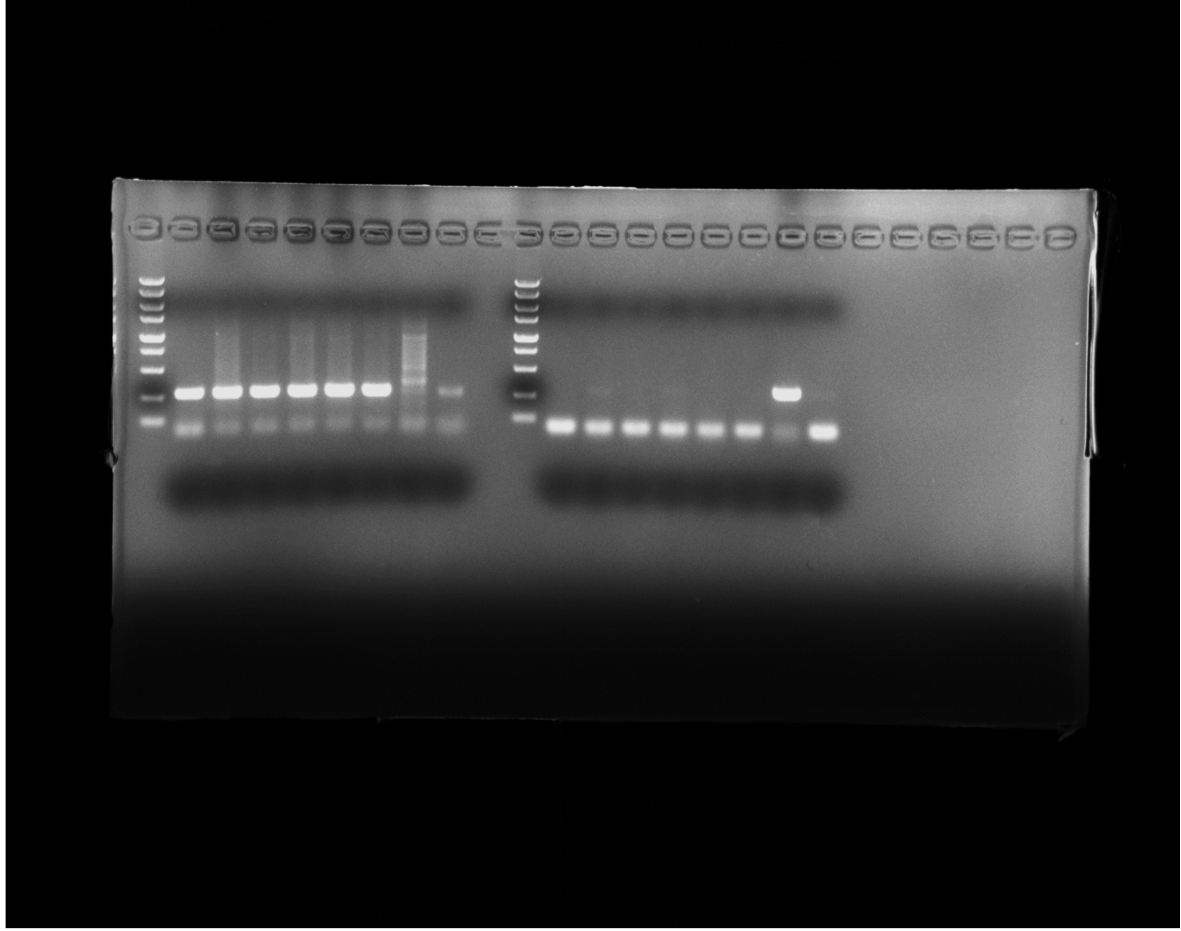

Supplement: Figure 3—figure supplement 1—source data 2. [file elife-92994-fig3-figsupp1-data2.zip › Figure 3ΓÇöfigure supplement 1A.tif]

Figure4—source data 1

Figure4C

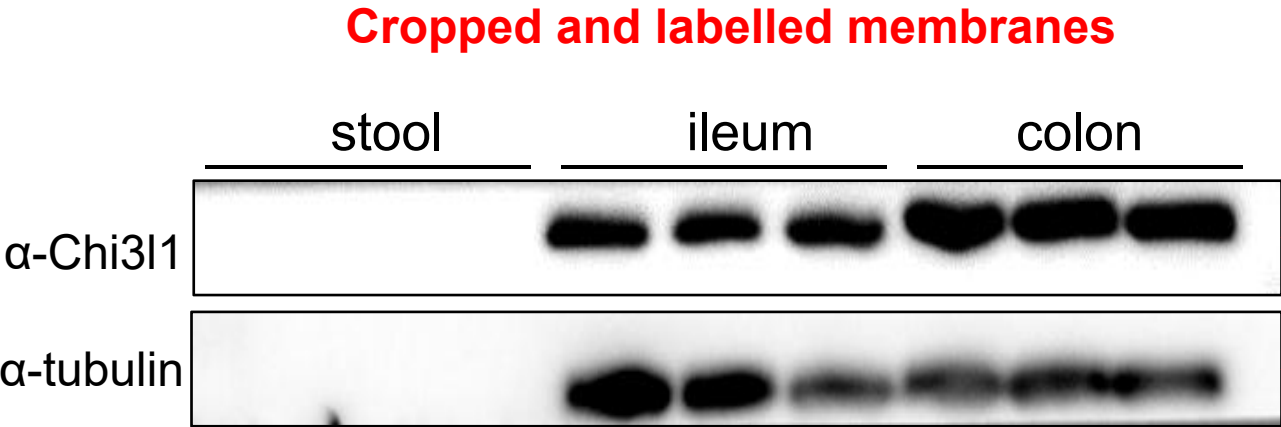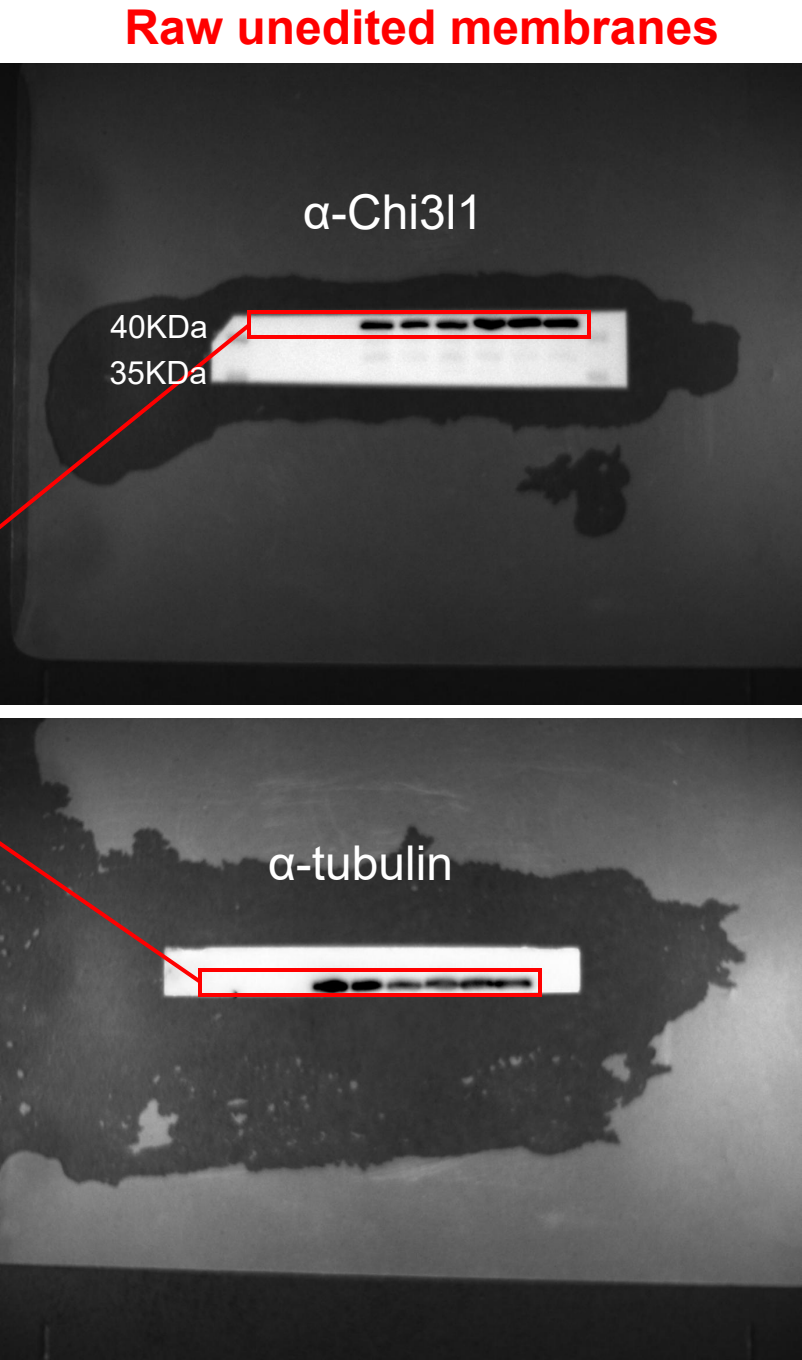

Figure4—source data 1

Figure4D

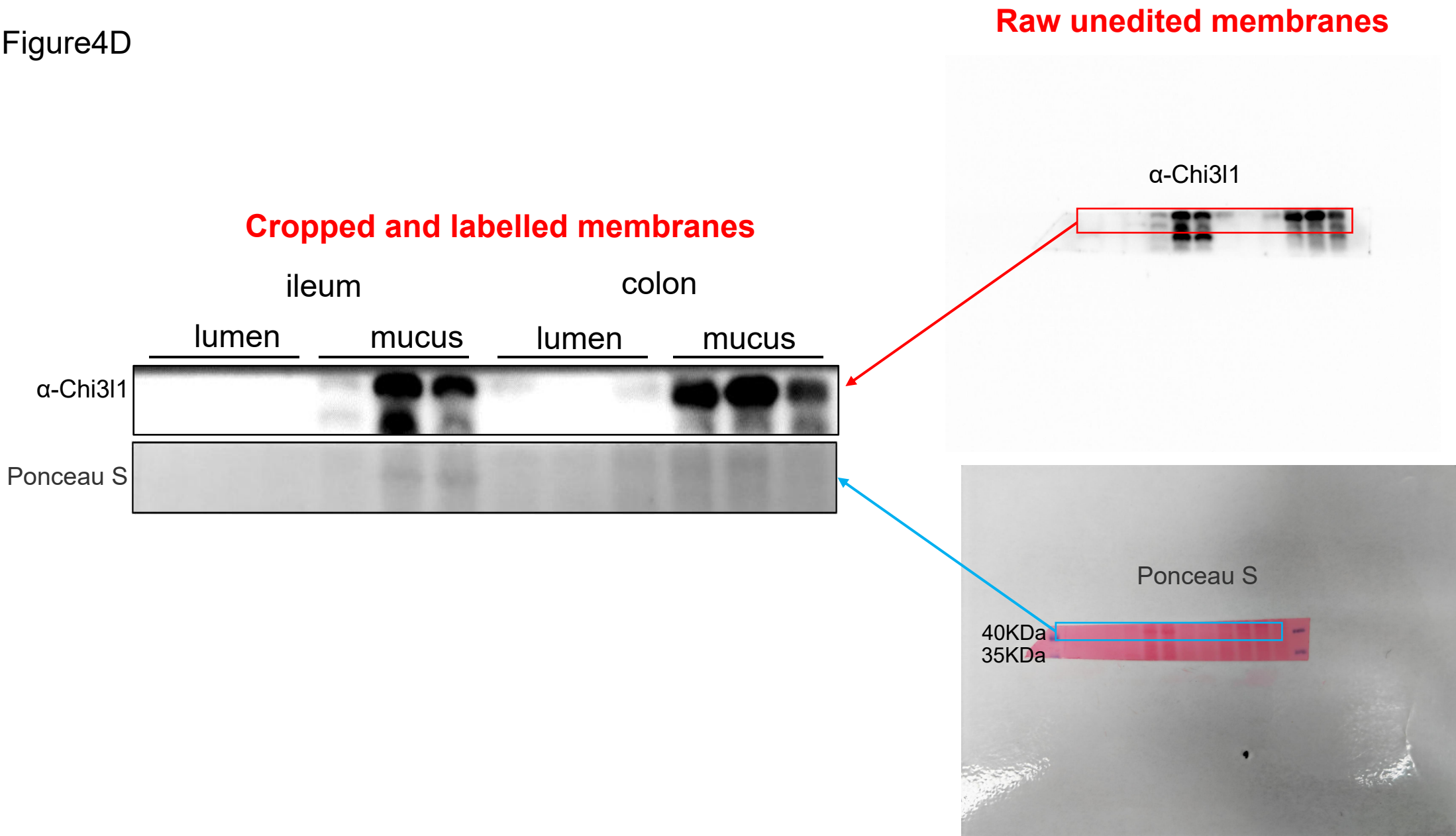

Supplement: Figure 4—source data 1. [file elife-92994-fig4-data1.zip › Figure 4-source data 1/Figure 4-source data 1.pdf]

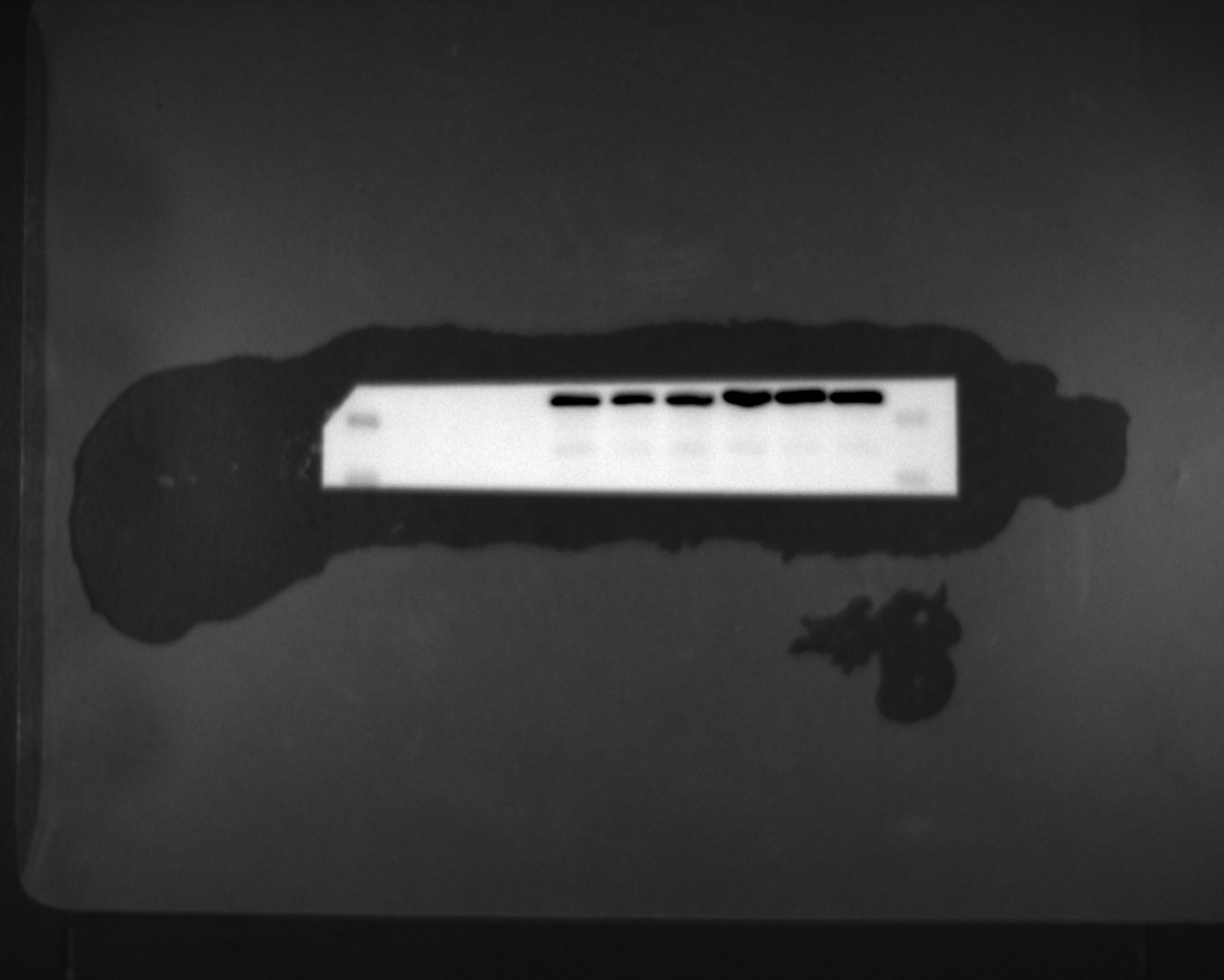

Supplement: Figure 4—source data 2. [file elife-92994-fig4-data2.zip › Figure 4-source data 2/Figure4C-1.tif]

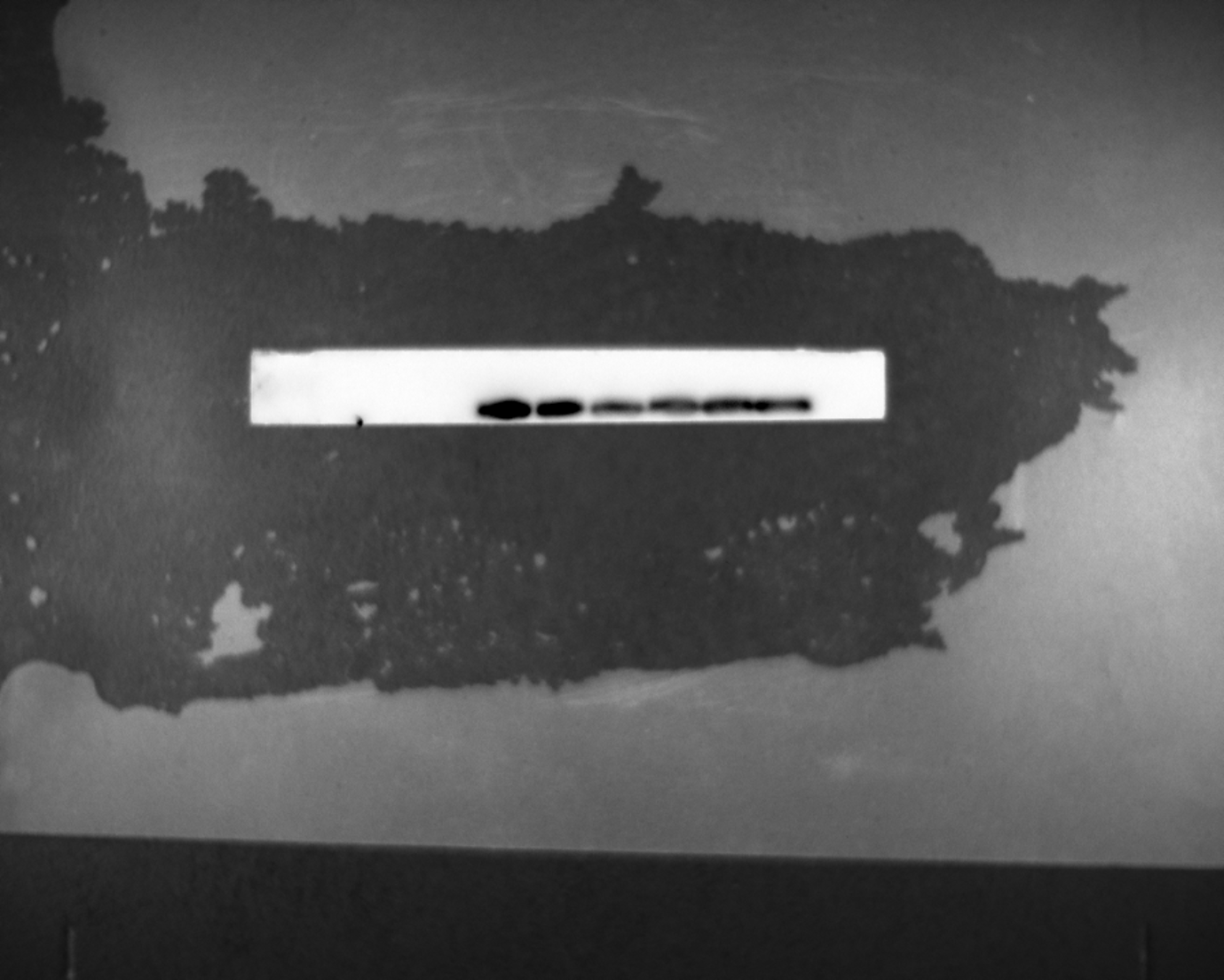

Supplement: Figure 4—source data 2. [file elife-92994-fig4-data2.zip › Figure 4-source data 2/Figure4C-2.tif]

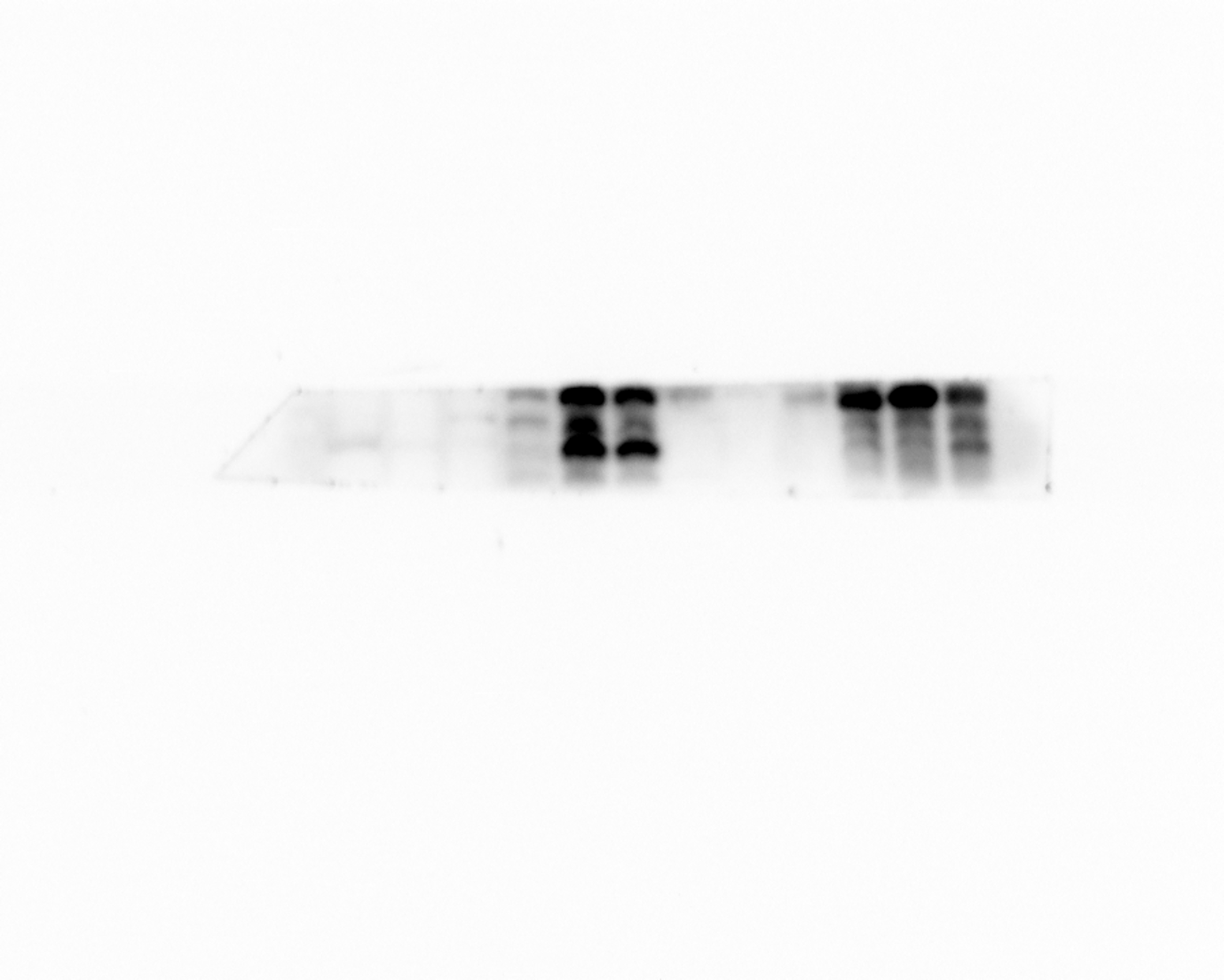

Supplement: Figure 4—source data 2. [file elife-92994-fig4-data2.zip › Figure 4-source data 2/Figure4D-1.tif]

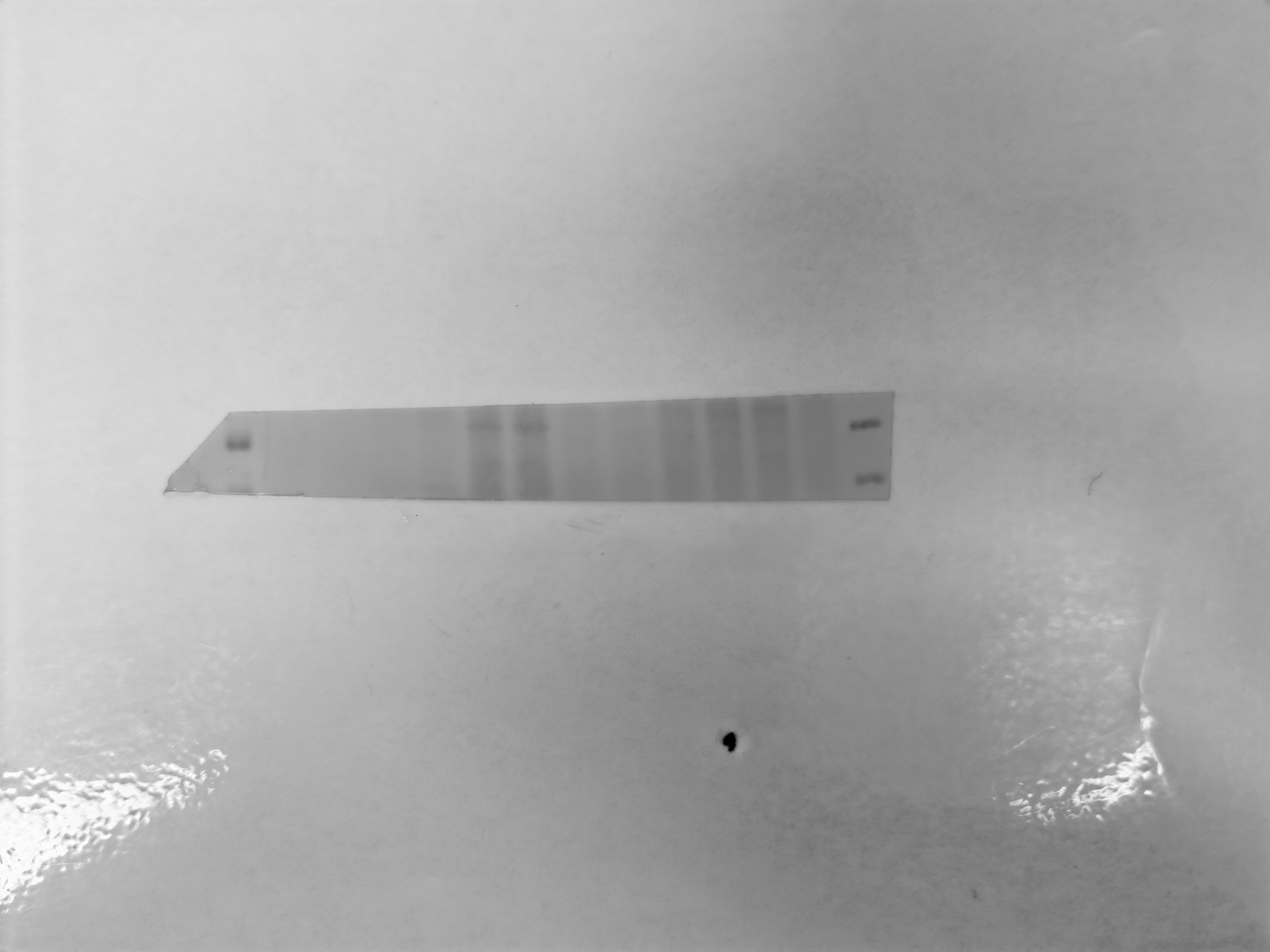

Supplement: Figure 4—source data 2. [file elife-92994-fig4-data2.zip › Figure 4-source data 2/Figure4D-2-1.jpg]

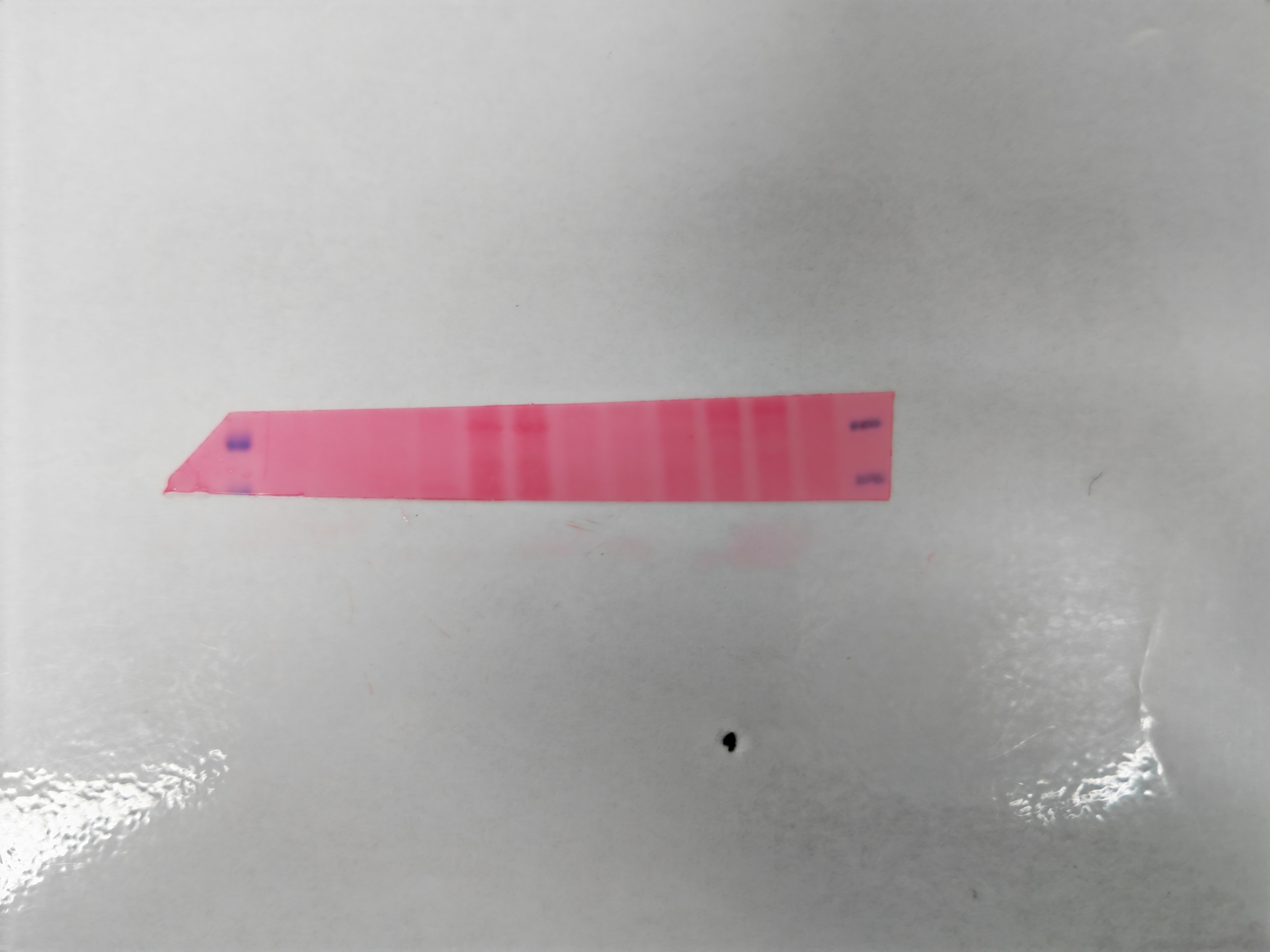

Supplement: Figure 4—source data 2. [file elife-92994-fig4-data2.zip › Figure 4-source data 2/Figure4D-2.jpg]
