# Supplementary material for: Peptidoglycan-Chi3l1 interaction shapes gut microbiota in intestinal mucus layer
Source: eLife. 2024 Oct 7;13:RP92994. doi: 10.7554/eLife.92994 (PMC11458176; doi:10.7554/eLife.92994)

Figure 2—Source Data 1

Figure 2B

Cropped and labelled membranes

Raw unedited membranes

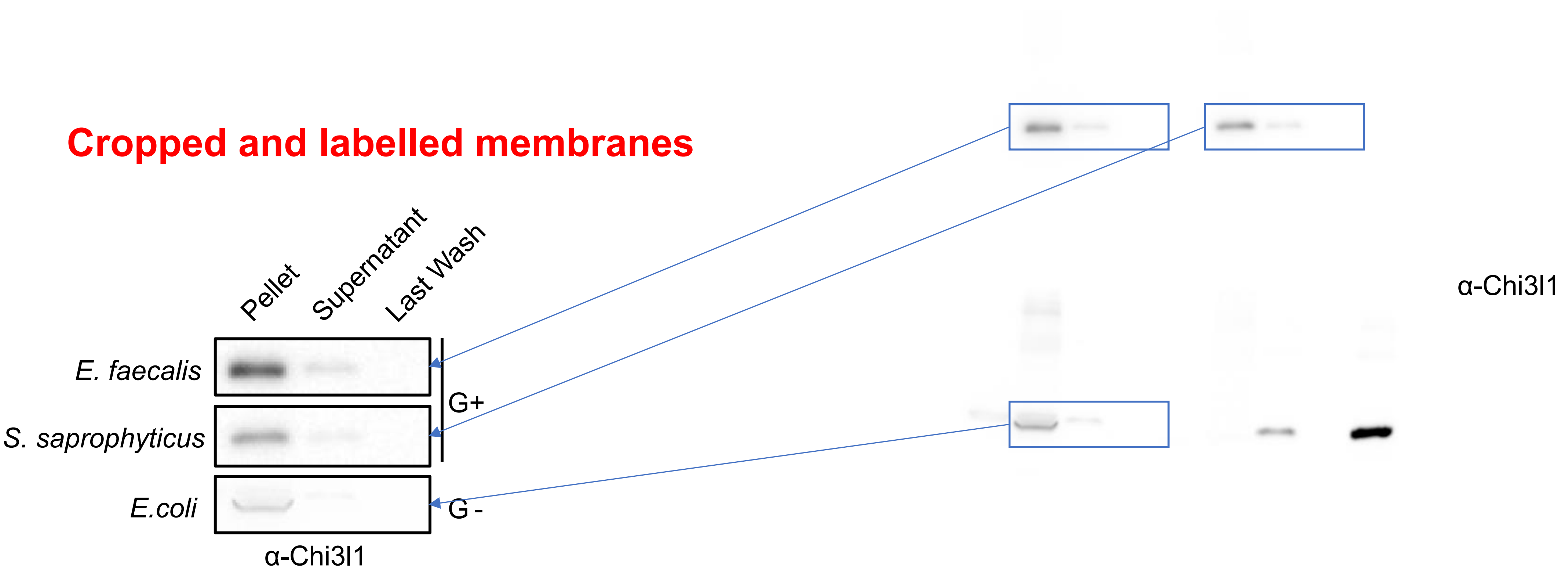

Figure 2—Source Data 1

Figure 2C

Cropped and labelled gels

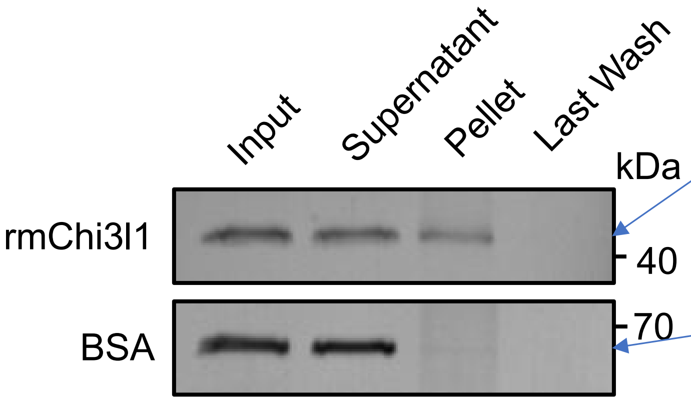

Raw unedited gels

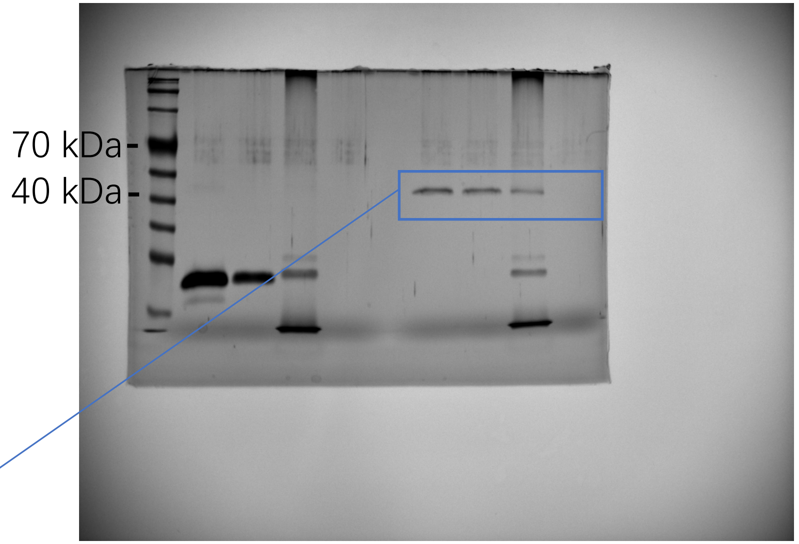

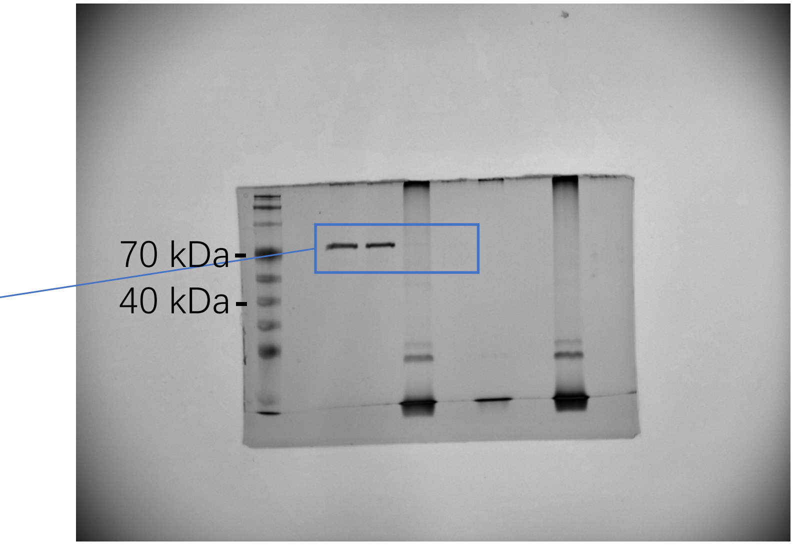

Figure 2—Source Data 1

Figure 2D

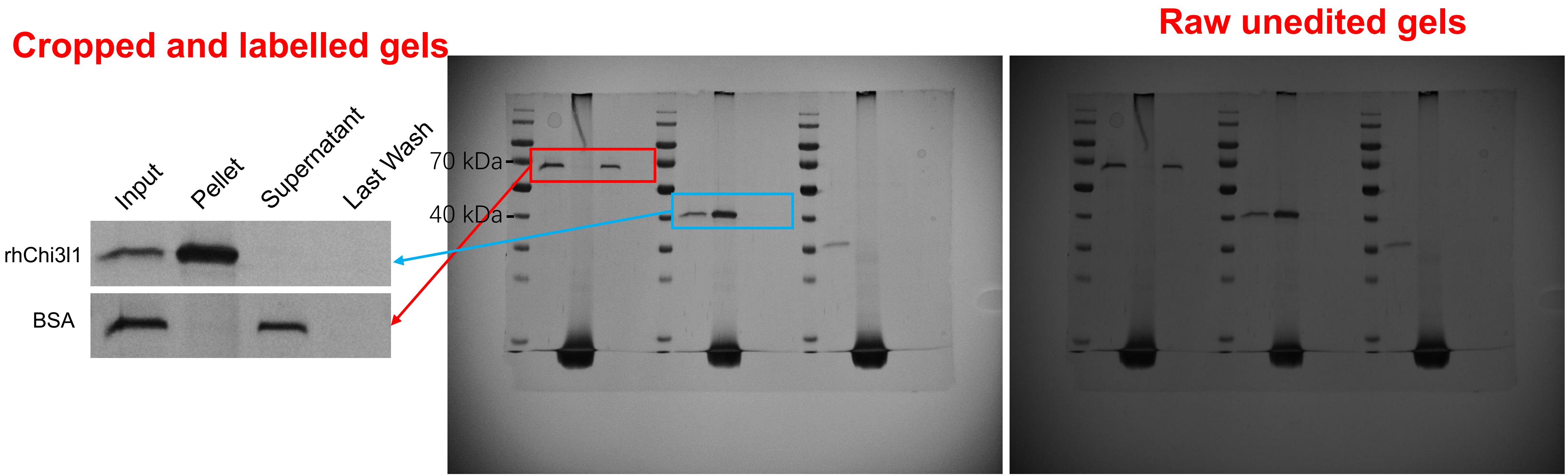

Figure 2—Source Data 1

Figure 2E

Cropped and labelled gels

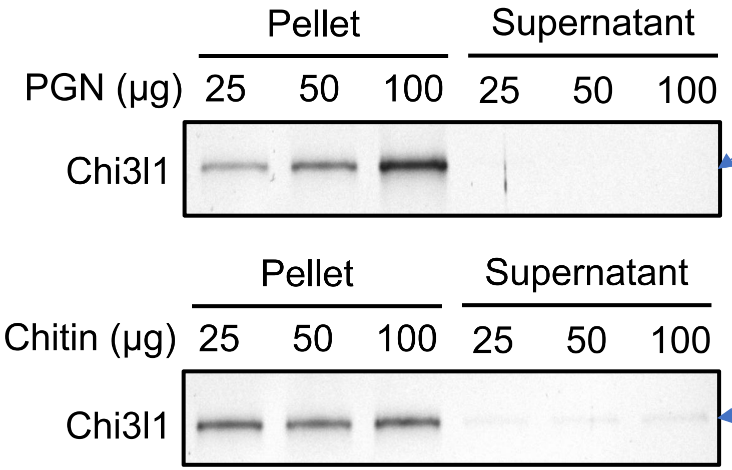

Raw unedited gels

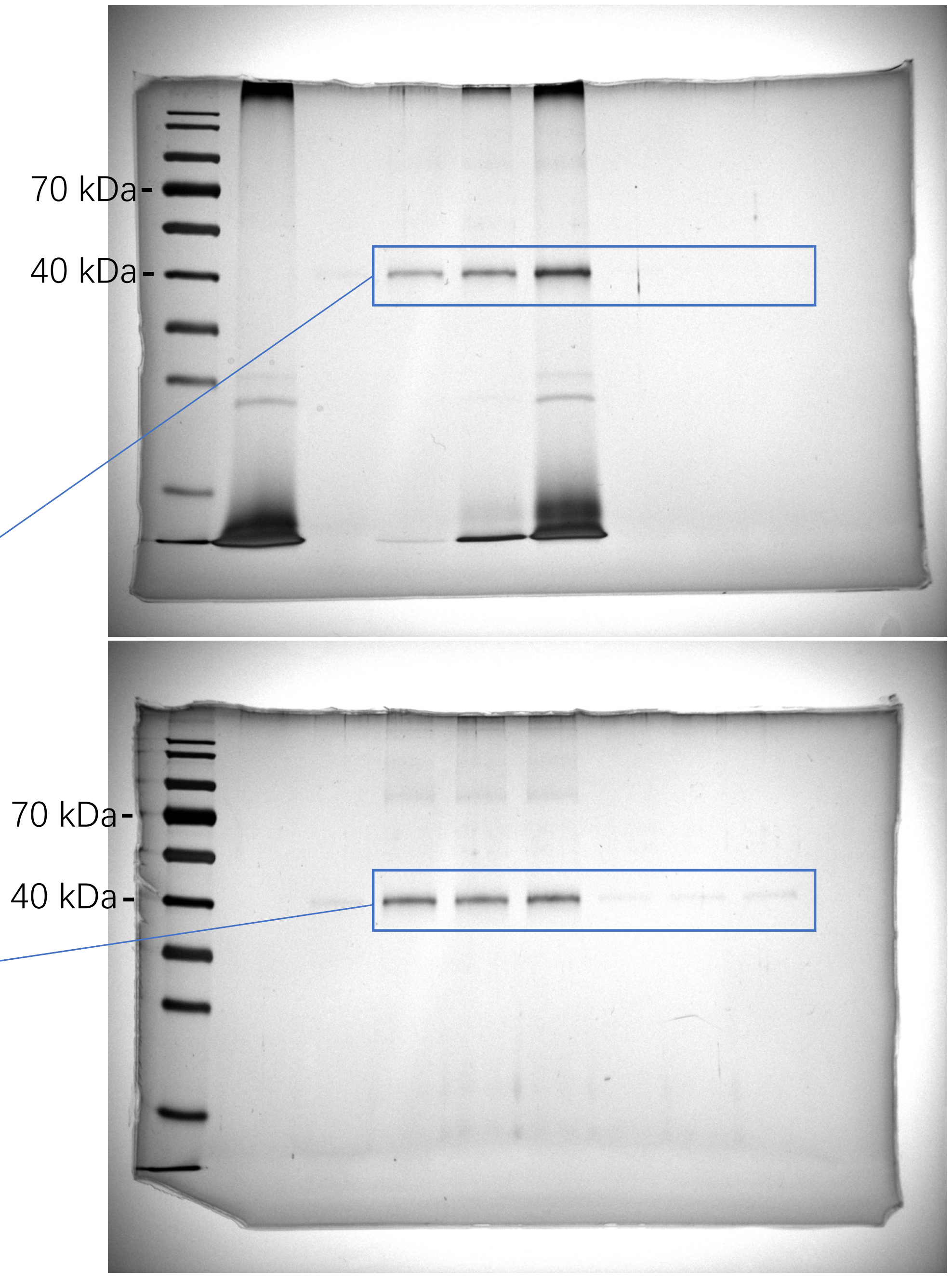

Supplement: Figure 2—source data 1. [file elife-92994-fig2-data1.zip › Figure 2-source data 1/Figure 2-source data 1.pdf]
