# Supplementary material for: Peptidoglycan-Chi3l1 interaction shapes gut microbiota in intestinal mucus layer
Source: eLife. 2024 Oct 7;13:RP92994. doi: 10.7554/eLife.92994 (PMC11458176; doi:10.7554/eLife.92994)

Figure 3—Source Data 2

Raw unedited gel

Figure 3—figure supplement 1A.

Cropped and labelled gel

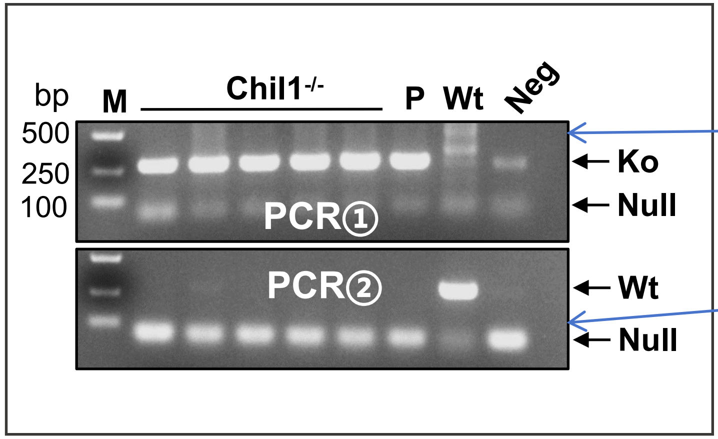

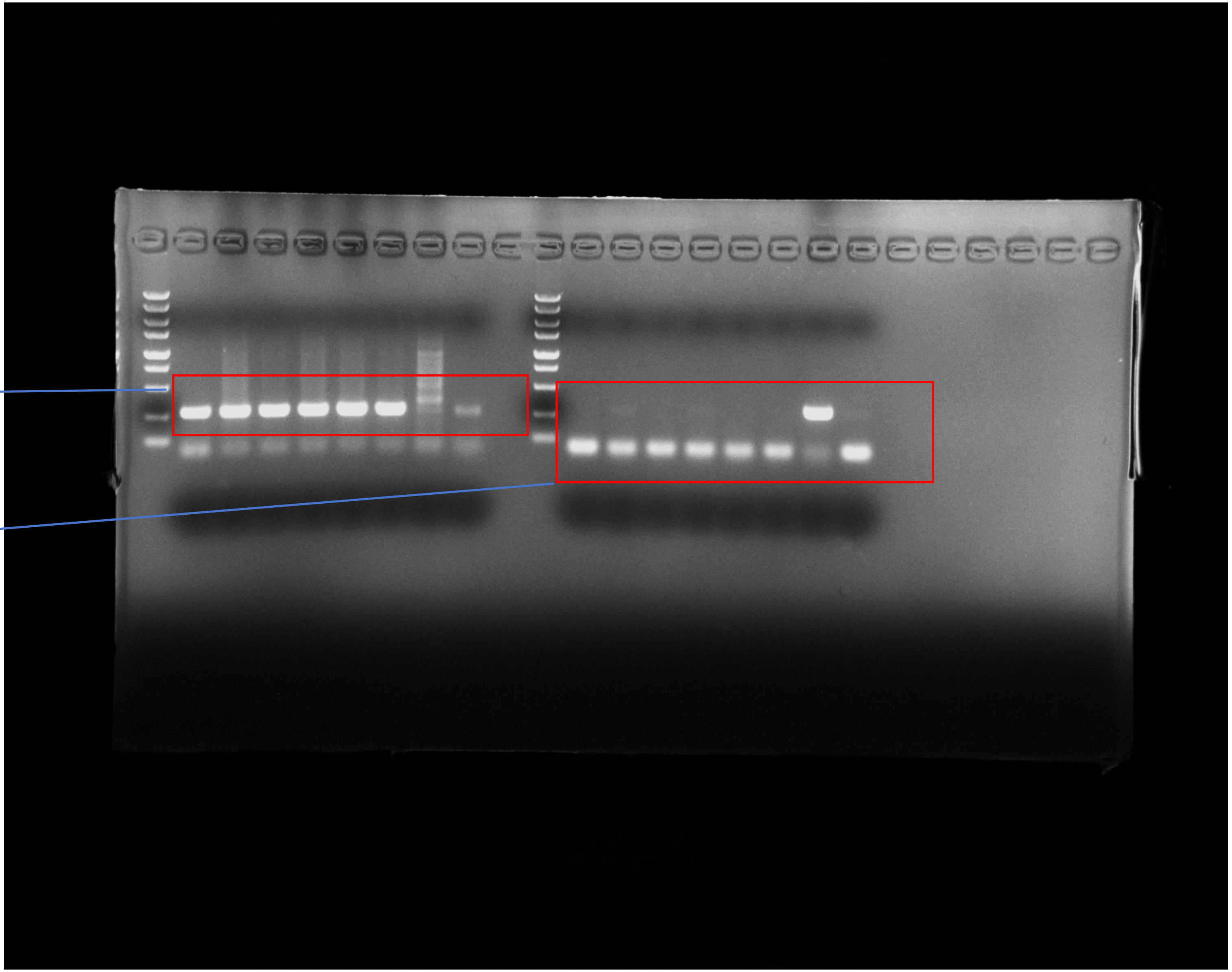

Figure 3—Source Data 2

Figure 3—figure supplement 1B.

Cropped and labelled gel

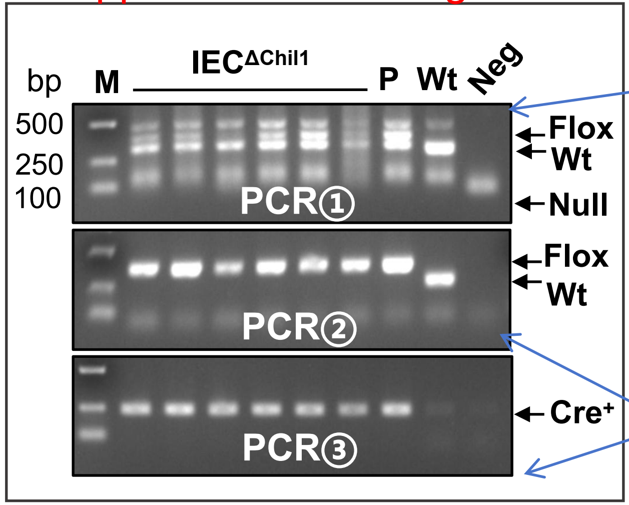

Raw unedited gel

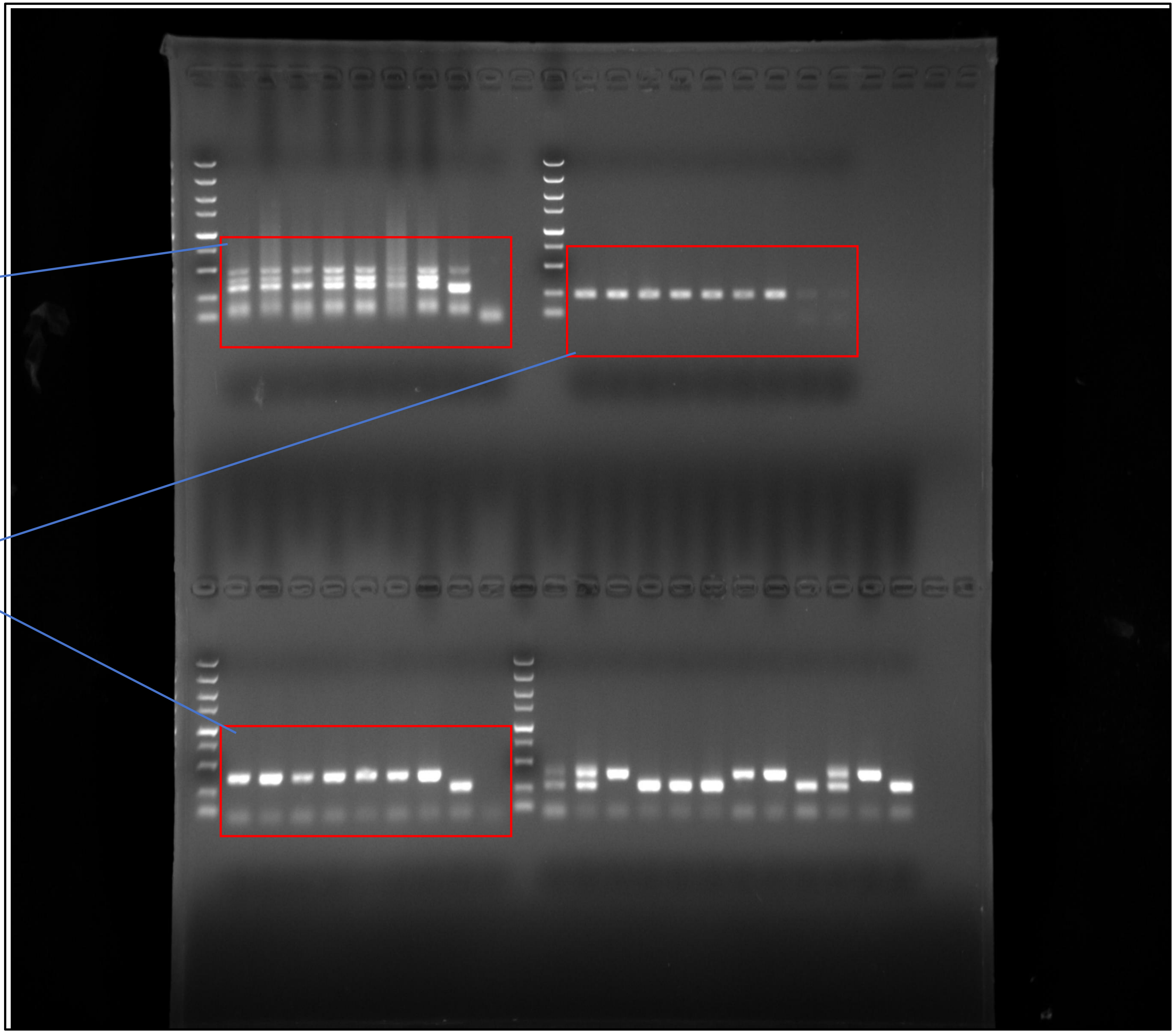

Supplement: Figure 3—figure supplement 1—source data 1. [file elife-92994-fig3-figsupp1-data1.zip › Figure 3ΓÇöfigure supplement 1ΓÇösource data 1.pdf]
